# Systematic Analysis of the *DOF* Gene Family in Flowering Chinese Cabbage (*Brassica campestris*) and the Role of BcDOF56 in Abiotic Stress Adaptation

**DOI:** 10.3390/biology15211940

**Published:** 2026-10-25

**Authors:** Jianguo Liu, Jiaxin Liu, Chunfeng Chen, Yukun Wang, Bin Wang, Huimin Feng, Shiwei Song, Yunna Zhu

**Affiliations:** 1 Guangdong Provincial Key Laboratory of Utilization and Conservation of Food and Medicinal Resources in Northern Region, College of Biology and Agriculture, Shaoguan University, Shaoguan 512005, China; jgliu@sgu.edu.cn (J.L.); wangyu_kun1@163.com (Y.W.); b_wang@sgu.edu.cn (B.W.); dxseed0708@163.com (H.F.); 2 College of Horticulture, South China Agricultural University, Guangzhou 510642, China; 3066128441jx@gmail.com (J.L.); ccf18864835013@stu.scau.edu.cn (C.C.)

**Keywords:** *DOF* gene family, flowering Chinese cabbage (*Brassica campestris*), BcDOF56, abiotic stress, antioxidant defense

## Abstract

DNA-binding with One Finger (DOF) transcription factors play crucial roles in modulating plant growth, development, and environmental stress responses. However, the DOF family in flowering Chinese cabbage (*Brassica campestris*) remains poorly characterized. Here, 67 *BcDOF* genes were identified in *B. campestris*, distributed nonuniformly across ten chromosomes and classified into nine subfamilies. Structural analyses revealed that all BcDOF proteins harbor a conserved zinc-finger DOF (zf-DOF) domain, with members within individual subfamilies generally sharing similar motif architectures and exon–intron structures. Evolutionary analyses indicated that whole-genome triplication and segmental duplication were the major drivers of *BcDOF* family expansion, while purifying selection contributed to the evolutionary conservation of duplicated gene pairs. Promoter analysis revealed abundant *cis*-acting elements related to hormone signaling and abiotic stress responses. Reverse transcription quantitative real-time PCR (RT-qPCR) further demonstrated that many *BcDOF* genes responded to heat and osmotic stresses. Among them, *BcDOF56* was markedly induced by both heat and drought and localized to the nucleus. Heterologous overexpression of *BcDOF56* in *Arabidopsis* significantly improved heat and drought tolerance, as indicated by reduced thiobarbituric acid reactive substances (TBARS) accumulation and elevated superoxide dismutase (SOD) activity relative to wild-type (WT) plants. Collectively, these findings advance our understanding of the evolutionary and functional divergence of the *BcDOF* family and highlight *BcDOF56* as a potential target gene for improving abiotic stress tolerance in *B. campestris*.

## Simple Summary

DOF proteins are plant-specific transcriptional regulators that contain a conserved zinc-finger DOF domain. However, the characteristics and functions of the *DOF* gene family in flowering Chinese cabbage (*Brassica campestris*) remain largely unexplored. Here, we identified 67 *BcDOF* genes in *B. campestris*, which showed conserved protein domain architectures and gene structures. *BcDOF* promoters harbor numerous *cis*-acting elements associated with hormone signaling and environmental stress responses. Notably, BcDOF56 was localized to the nucleus, and its expression was significantly induced by heat and osmotic stresses. Heterologous overexpression of *BcDOF56* enhanced tolerance to heat and drought, possibly by promoting antioxidant defense. These findings provide valuable insights into the stress-responsive roles of *BcDOFs* and suggest that *BcDOF56* is a potential target gene for breeding stress-tolerant *B. campestris* varieties.

## 1. Introduction

The DNA-binding with One Finger (DOF) family is a plant-specific group within the zinc-finger superfamily. Following the isolation of *ZmDOF1* from maize (*Zea mays*) in 1993 [1], DOF homologs have been characterized in organisms ranging from algae to higher plants [2,3,4,5]. DOF proteins, generally ranging from 200 to 400 amino acids in size, are structurally defined by a highly conserved N-terminal DOF domain of approximately 52 residues. This domain forms a single C_2_-C_2_ zinc finger with the consensus sequence CX_2_CX_21_CX_2_C [3]. In contrast, the C-terminal segment harbors a highly divergent transcriptional regulatory domain, which serves as the primary structural basis for the functional diversification of DOF proteins [6].

DOF transcription factors are highly conserved across spermatophytes and early terrestrial plants [7], with an evolutionary origin predating the divergence of *Chlorophyta* and embryophytes [8]. The green alga *Chlamydomonas reinhardtii* retains only a single *DOF* homolog, indicating that the extant *DOF* family most likely originated from this ancestral gene copy [4]. Subsequent extensive gene duplications drove the expansion of the *DOF* family during angiosperm diversification, facilitating plant adaptation to dynamic terrestrial environments [4]. To date, *DOF* family members have been characterized in numerous plant species, with gene copy number varying considerably. In monocots, for instance, genome-wide surveys have reported 30, 96, and 27 *DOF* genes in rice (*Oryza sativa*) [9], wheat (*Triticum aestivum*) [10], and orchid (*Cymbidium goeringii*) [11], respectively. In dicots, 36 *DOF* genes have been characterized in *Arabidopsis thaliana* [12], 33 in tomato (*Solanum lycopersicum*) [13], 76 in Chinese cabbage (*Brassica rapa*) [14], 117 in rapeseed (*Brassica napus*) [15], 44 in *Populus trichocarpa* [16], and 75 in tobacco (*Nicotiana tabacum*) [17]. This pronounced divergence in copy number highlights the contribution of *DOF* expansion to lineage-specific functional diversification, enabling plants to cope with multifaceted environmental stresses [4].

Functionally, DOF transcription factors participate in multiple physiological processes, such as seed germination [18], leaf senescence and floral morphogenesis [19,20], and fruit ripening [21]. In *Arabidopsis*, AtDOF6 represses seed germination by interacting with AtTCP14 [18], whereas AtDOF2.1 promotes leaf senescence by activating jasmonic acid biosynthesis [20]. Overexpression of rapeseed *BnCDF1* (cycling DOF factor 1) in *Arabidopsis* delays flowering by regulating the photoperiod pathway [22], while AtDOF4.7 accelerates floral organ abscission by inhibiting cell wall hydrolase activity [19]. In banana (*Musa acuminata*), MaDOF23 negatively regulates ethylene biosynthesis to modulate fruit ripening [21]. Beyond these developmental roles, DOF proteins serve as critical integrators of stress signaling, mediating plant tolerance to environmental stresses, including extreme temperatures, drought, salinity, and pathogen infection [23]. For instance, the transcript abundance of *AtDOF5.8* is significantly induced by drought and salt stresses in *Arabidopsis* [24]. In *B. rapa*, most *BraDof* genes exhibit distinct transcriptional changes under abiotic stress conditions: the transcript levels of *BraDof003*, *BraDof045*, and *BraDof053* are significantly upregulated under extreme temperature stress, whereas *BraDof072* is strongly repressed; furthermore, drought and salinity treatments induce the expression of *BraDof045*, *BraDof053*, and *BraDof074* [14]. In walnut (*Juglans regia*), JrDOF3 improves thermotolerance through targeted activation of heat shock proteins [25]. Both rapeseed BnCDF1 and rice OsDOF1 enhance plant cold tolerance [22,26]. Moreover, *PsDOF* genes in *Populus simonii* are consistently upregulated across multiple tissues under diverse stress conditions [27]. Notably, a recent study in tobacco revealed opposing functions among DOF family members under cold stress: NtDOF40 enhances cold tolerance, while NtDOF63 acts as a negative regulator [17]. Collectively, these studies establish DOF transcription factors as critical integrators of plant development and stress adaptation. However, DOF members exhibit pronounced functional divergence across plant species, executing distinct or even antagonistic biological roles.

Flowering Chinese cabbage (*Brassica campestris* L. ssp. *chinensis* var. *utilis* Tsen et Lee), a variant of *B. rapa*, is an economically and nutritionally vital stalk vegetable widely cultivated across southern China [28,29]. Unlike most Brassicaceae crops that require low-temperature vernalization to initiate bolting, flowering Chinese cabbage exhibits vernalization-independent development [30,31]. However, biotic and abiotic stress conditions often disrupt normal stalk development, causing substantial yield losses and quality deterioration that severely constrain year-round production of flowering Chinese cabbage [29]. Global climate change further threatens crop productivity, underscoring the urgent need to explore stress-tolerant genetic resources for crop improvement [32]. Although DOF gene families have been extensively investigated in various plant species, systematic characterization and functional analysis of DOF members remain lacking in flowering Chinese cabbage. The recently released reference genome of this crop provides a valuable genomic resource for systematic gene family studies [30].

Here, we systematically surveyed and profiled the *DOF* gene family across the genome of flowering Chinese cabbage, elucidating their chromosomal localization, gene structures, phylogenetic relationships, syntenic patterns, and *cis*-regulatory elements. We further investigated the organ-specific expression profiles of *BcDOFs* and explored their responses to heat and drought stresses. Collectively, these findings establish a comprehensive framework for deciphering BcDOF biological functions and provide promising candidate genes for breeding stress-resilient cultivars.

## 2. Materials and Methods

### 2.1. Identification and Chromosome Localization of DOF Gene Family in Brassica campestris

The genome sequences and annotations of *B. campestris* were retrieved from the China National GeneBank DataBase (CNGBdb) (https://db.cngb.org/; accessed on 10 June 2025), based on data from Li et al. [30]. A total of 36 *Arabidopsis* DOF proteins were obtained from TAIR (https://www.arabidopsis.org/; accessed on 10 June 2025) and served as queries for BLASTP searches against the *B. campestris* genome (E-value < 1 × 10^−5^) with the BLAST GUI Wrapper in TBtools-II v.2.482 [33]. In parallel, the Advanced HMMER Search module in TBtools-II v.2.482 was employed to screen candidate DOF members from this genome using the zinc-finger DOF (zf-DOF) domain model (Pfam: PF02701). Non-redundant candidates pooled from both searches were subsequently validated for intact, characteristic DOF motifs using the NCBI Batch CD-Search tool (https://www.ncbi.nlm.nih.gov/Structure/bwrpsb/bwrpsb.cgi, accessed on 10 June 2025), thereby establishing the final catalog of BcDOF members.

Physicochemical properties of the predicted DOF proteins were analyzed with the ExPASY ProtParam tool (https://web.expasy.org/protparam/, accessed on 10 June 2025). Subcellular localization of DOF proteins was inferred using DeepLoc-2.0 (https://services.healthtech.dtu.dk/services/DeepLoc-2.0/; accessed on 10 June 2025), and their chromosomal positions were visualized using TBtools-II v.2.482 [33].

### 2.2. Phylogenetic Analysis and Subfamily Classification of DOF Proteins

To elucidate evolutionary relationships and subfamily classifications, DOF protein sequences from *B. campestris* and *Arabidopsis* were aligned using ClustalW under default parameter settings. MEGA 7.0 was employed to construct a neighbor-joining (NJ) tree supported by 1000 bootstrap replicates, followed by visualization in FigTree v1.4.4.

In parallel, to explore cross-species evolutionary dynamics, a multi-species phylogenetic tree covering ten representative plant species was constructed via OrthoVenn3 (https://orthovenn3.bioinfotoolkits.net/home, accessed on 12 July 2025) [34]. The species included *C. reinhardtii*, *Z. mays*, *P. trichocarpa*, *O. sativa*, *A. thaliana*, *S. lycopersicum*, *Cucumis sativus*, *B. rapa*, *B. napus*, and *B. campestris*. Orthologous clusters were identified using the default OrthoMCL algorithm, and the species phylogeny was reconstructed using the maximum likelihood (ML) method under the JTT+CAT evolutionary model. The DOF members previously identified in *B. rapa* [14] were manually refined; two putative sequences (Bra002057 and Bras007632) were excluded due to their high sequence homology to *Arabidopsis* Syntaxin of Plant 21 and Auxin Response Factor 18, leaving 74 authentic BraDOF members for downstream analysis. Subfamily demarcations were assigned according to established classifications and visualized using Adobe Illustrator CC 2019 (Adobe Inc., San Jose, CA, USA).

### 2.3. Orthology and Synteny Analyses of BcDOFs

Genome sequences and corresponding annotations of *Arabidopsis*, *B. rapa*, and *B. napus* were retrieved from TAIR (TAIR9), BRAD (Brara_Chiifu_v1.5), and GenoScope (*Brassica_napus*.annotation_v5), respectively. Orthologous gene clusters spanning these three species alongside *B. campestris* were identified and visualized using OrthoVenn3 server (https://orthovenn3.bioinfotoolkits.net/home, accessed on 4 July 2025) [34].

Within *B. campestris*, gene duplication events were identified via the One Step MCScanX-Super Fast plugin and mapped using the Advanced Circos module in TBtools-II v.2.482 [33]. Additionally, interspecific syntenies of *DOF*s among *Arabidopsis*, *B. campestris*, *B. rapa*, and *B. napus* were characterized and visualized using the MCScanX-Super Fast plugin and the Multiple Synteny Plot module, respectively. The nonsynonymous (Ka) and synonymous (Ks) substitution rates were determined for orthologous gene pairs via the Simple Ka/Ks Calculator module. The divergence time (T) of paired orthologs was estimated via the equation T = Ks/(2λ), where λ = 1.5 × 10^−8^ substitutions per site per year, a neutral mutation rate previously calibrated for *B. rapa* [35].

### 2.4. Analysis of Conserved Motifs and Gene Structures of BcDOFs

Conserved motifs within BcDOFs were analyzed using the MEME Suite (https://meme-suite.org/meme/; accessed on 20 September 2025) with a maximum of 15 motifs. Their gene structural characteristics were visualized using TBtools-II v.2.482.

### 2.5. Profiling of Cis-Acting Elements in BcDOF Promoters

The 2000 bp genomic regions upstream of all *BcDOF* genes were retrieved from the *B. campestris* genome to predict putative *cis*-acting elements via the PlantCARE database (https://bioinformatics.psb.ugent.be/webtools/plantcare/html/; accessed on 25 September 2025). The identified elements were categorized based on their putative function and displayed with TBtools-II v.2.482.

### 2.6. Expression Profiling of BcDOFs Across Organs and Under Abiotic Stress

To characterize the organ-specific expression profiling of *BcDOFs*, publicly available RNA-seq datasets (SRP427920) derived from *B. campestris* cultivar ‘49 Caixin’ were analyzed. FPKM (fragments per kilobase of exon model per million mapped fragments) values were obtained across seven organs: roots, stems, young leaves, mature leaves, senescent leaves, flowers, and siliques. Normalized FPKM values were used to construct a heatmap.

For abiotic stress treatments, *B. campestris* cultivar ‘Youlv 501’ served as experimental material. Seedlings were grown in a controlled environmental chamber (T: 25 ± 2 °C; relative humidity: 75%; light intensity: 100 μmol∙m^−2^∙s^−1^; photoperiod: 16 h light/8 h dark) for 14 days. Uniform seedlings were transplanted into half-strength Hoagland solution and cultured for 7 days. At the five-leaf stage, seedlings were transferred to distinct stress regimes: heat treatment was applied at 40 °C in a constant-temperature incubator, while osmotic stress was simulated by supplementing the nutrient solution with 10% (*w*/*v*) polyethylene glycol 6000 (PEG 6000). Roots and leaves were harvested at 0, 3, 24, and 48 h after treatment. All assays were conducted with three biological replicates per condition.

### 2.7. Reverse Transcription Quantitative Real-Time PCR (RT-qPCR) Analysis

Total RNA was extracted using Eastep^®^ Super Total RNA Extraction Kit (Promega, Beijing, China), and first-strand cDNA was synthesized using GoScript™ Reverse Transcriptase Kit (Promega). Reverse transcription quantitative real-time PCR (RT-qPCR) was conducted on a CFX Connect^TM^ system (Bio-Rad, Hercules, CA, USA) with SYBR^®^ Green Premix Pro Taq HS qPCR Kit (Accurate Biology, Changsha, China). All RT-qPCR primers are detailed in Table S1. Relative gene expression was determined via the 2^−ΔΔCt^ method [36], with *BcGAPDH* serving as the internal reference gene.

### 2.8. Overexpression of BcDOF56 and Functional Analysis in Arabidopsis

The coding sequence (CDS) of *BcDOF56* was inserted into the overexpression vector pCAMBIA3301 and subsequently transformed into *Arabidopsis* ecotype Columbia-0 (Col-0) via an *Agrobacterium tumefaciens*-mediated floral dip protocol [37]. Putative transformants were screened across the T_1_, T_2_ and T_3_ generations under a uniform protocol via foliar spraying with a 1000-fold dilution of 20% Basta stock solution (Yuanye, Shanghai, China), followed by PCR of the *Bar* gene, and positive lines were further validated via RT-qPCR. Homozygous T_3_ progenies were used for subsequent function assays.

Both wild-type (WT) and transgenic seeds were germinated in a peat-vermiculite composite (3:1, *v*/*v*) supplied with quarter-strength Hoagland solution and cultivated under standard growth conditions for four weeks. For heat stress, seedlings were incubated at 40 °C for 60 h. For drought stress, watering was completely withheld for 15 days. Rosette leaves from both WT and transgenic lines were sampled to determine thiobarbituric acid reactive substances (TBARS) content and superoxide dismutase (SOD) activity. TBARS were quantified via the thiobarbituric acid colorimetric assay using a commercial kit (Cat. No. BC0020, Solarbio, Beijing, China), with malondialdehyde (MDA) as the predominant reactive constituent. SOD activity was assayed using a corresponding kit (Cat. NO. BC5160, Solarbio) following a previously described protocol [17].

### 2.9. Subcellular Localization Analysis of BcDOF56

The CDS of *BcDOF56* lacking the termination codon was inserted into the transient expression vector pBI121-GFP linearized with *Sma*I and *Xba*I. The recombinant pBI121-BcDOF56-GFP or pBI121-GFP was transformed into *Agrobacterium tumefaciens* GV3101. The nuclear localization signal (NLS)-mCherry served as a nuclear marker. *Agrobacterium* suspensions carrying NLS-mCherry together with either pBI121-GFP or pBI121-BcDOF56-GFP were co-infiltrated into *N. benthamiana* leaves. After infiltration for 72 h, fluorescence signals were visualized using an Axio Imager D2 microscope (Zeiss, Jena, Germany).

### 2.10. Statistical Analysis

Data are presented as the mean ± standard deviation (SD) (*n* = 3). Treatment differences were analyzed via one-way ANOVA followed by Tukey’s Honestly Significant Difference (HSD) test in SPSS v21.0 (IBM, Armonk, NY, USA), and lowercase letters denote significant differences at *p* < 0.05. Pairwise comparisons between WT and overexpression lines were evaluated via a two-tailed Student’s *t*-test in SPSS v21.0. Asterisks denote statistical significance: * *p* < 0.05, ** *p* < 0.01, and *** *p* < 0.001.

## 3. Results

### 3.1. Identification and Characterization of the BcDOF Gene Family in B. campestris

**Figure 1:**
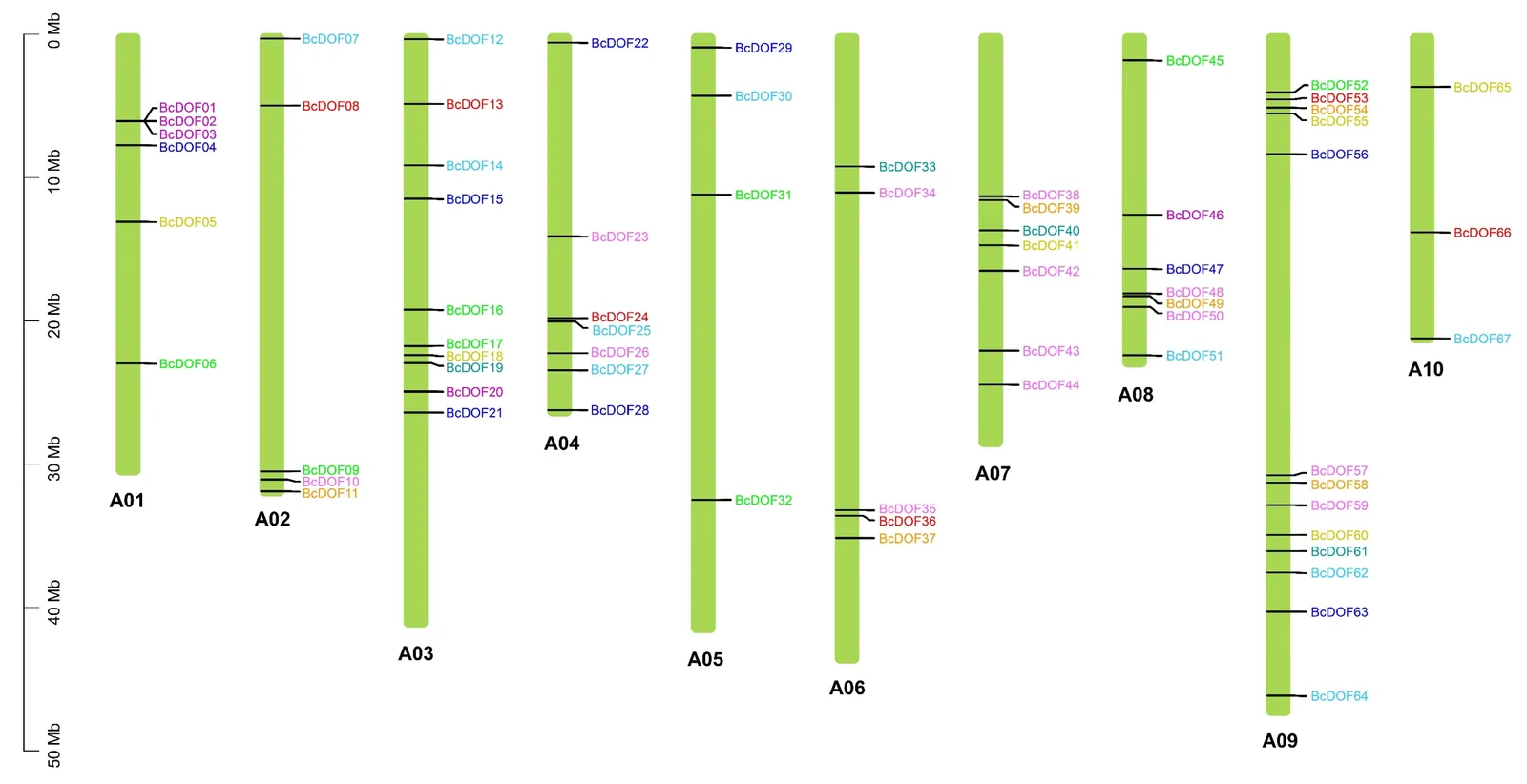
Chromosomal localization and phylogenetic clustering of *BcDOF* genes in *Brassica campestris*. Each vertical bar represents an individual chromosome. Physical position of each gene is annotated, and relative chromosome length in megabases (Mb) is shown by the scale bar. Genes shown in the same color belong to the same phylogenetic cluster.

To identify candidate DOF family members in the *B. campestris* genome, we performed BLASTP searches using 36 *Arabidopsis* DOF proteins retrieved from TAIR. Following structural validation of the signature zf-DOF domain (Pfam: PF02701), a final set of 67 BcDOF candidates was identified and sequentially designated as BcDOF01 to BcDOF67 according to their physical coordinates on chromosomes (Figure 1; Table 1).

Chromosomal mapping revealed that all 67 *BcDOF* genes were nonuniformly distributed across ten chromosomes of *B. campestris*. Chromosome A09 contained the largest number (13 members, *BcDOF52*–*BcDOF64*), followed by A03 (10, *BcDOF12*–*BcDOF21*) and A01 (6, *BcDOF01*–*BcDOF06*). Chromosomes A04, A07, and A08 each harbored seven genes; A02 and A06 each had five genes; and A05 and A10 carried four and three genes, respectively.

The detailed physicochemical properties of all predicted BcDOF proteins are summarized in Table 1. Polypeptide lengths spanned from 148 (BcDOF20) to 482 amino acids (BcDOF23), corresponding to molecular weights ranging from 17.03 to 52.17 kDa. Predicted theoretical isoelectric points (pI) spanned from 4.73 (BcDOF33) to 9.74 (BcDOF20). Most BcDOF proteins displayed instability indices over 40, indicating their relative instability. All BcDOF proteins exhibited negative grand average of hydropathy (GRAVY) values, varying from −0.323 to −1.092, confirming their hydrophilic characteristics. Furthermore, all BcDOF proteins were predicted to be localized to the nucleus, consistent with their function as transcription factors.

### 3.2. Phylogenetic Analysis of the BcDOF Gene Family

**Figure 2:**
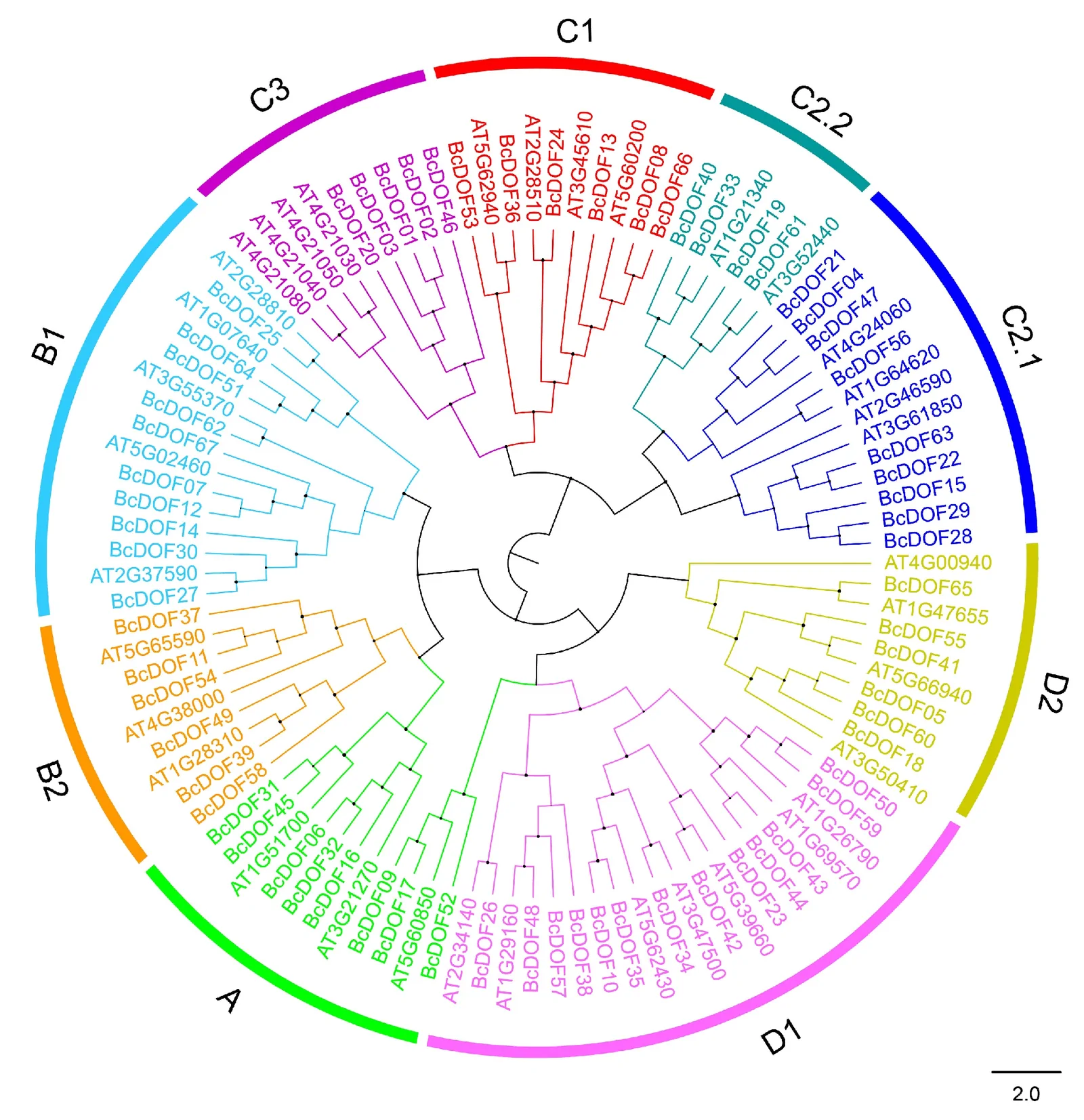
Phylogenetic relationships of DOF proteins from *B. campestris* and *Arabidopsis*. The tree was constructed using the neighbor-joining (NJ) algorithm in MEGA 7.0, supported by 1000 bootstrap replicates. A total of 67 BcDOF and 36 AtDOF proteins were included in this analysis.

To elucidate the classification and evolutionary relationship of DOF transcription factors, a phylogenetic tree was constructed in MEGA 7.0, including 67 BcDOF proteins from *B. campestris* and 36 AtDOFs from *Arabidopsis* (Figure 2). Tree topology assigned all DOF members into four major clades (Groups A–D). Groups B, C, and D were each further split into specific subfamilies: B_1_/B_2_, C_1_, C_2.1_, C_2.2_, C_3_, and D_1_/D_2_. Among the major clades, Group C contained the highest proportion of BcDOF members (35.82%, 24 genes), followed by Group D (28.36%, 19 genes). Among the nine subgroups, Subgroup D_1_ was the largest (19.40%), whereas Subgroup C_2.2_ was the smallest, representing only 5.97% of total BcDOF members (Figure 2).

**Figure 3:**
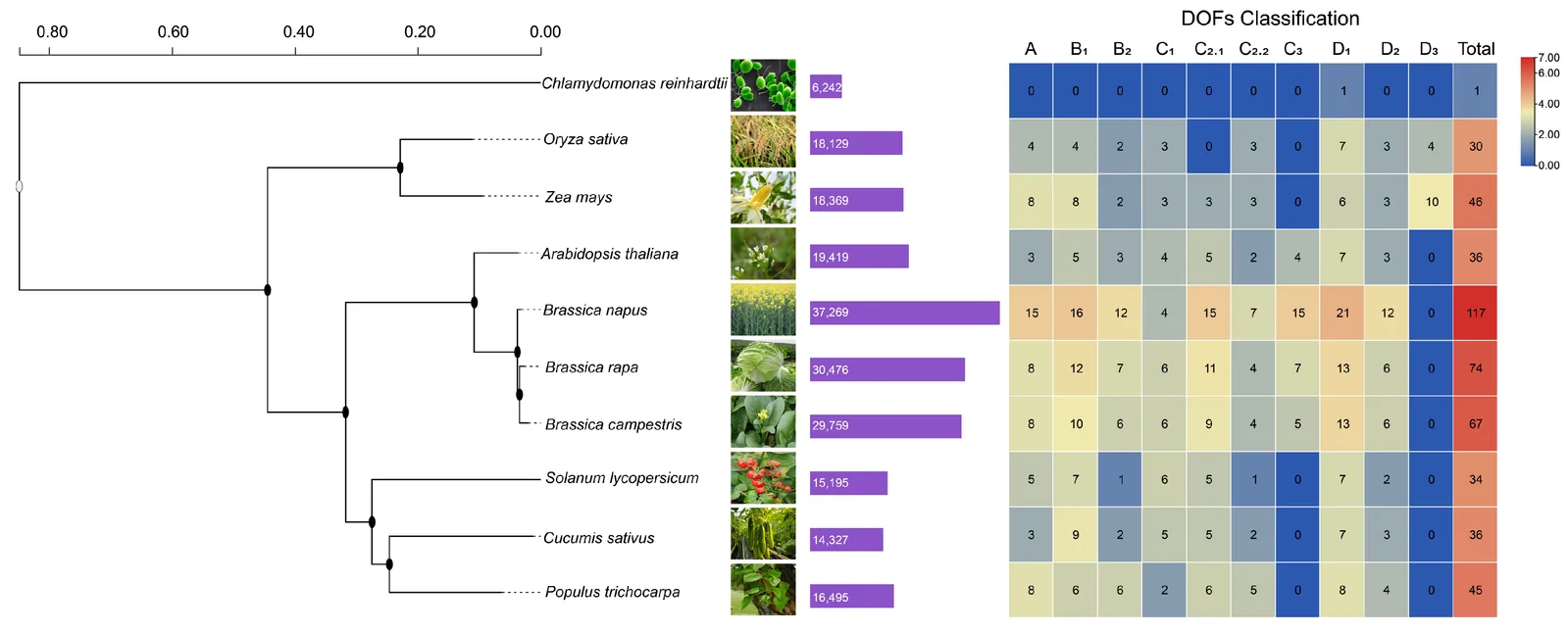
Comparative analysis of DOF gene families across different plant species. The values in the purple boxes represent the total number of orthogroups in each species, while those in colored boxes indicate the number of DOF members.

To elucidate the evolutionary characteristics of DOF proteins, a comparative phylogeny was reconstructed across ten representative plant species, covering the green alga (*C. reinhardtii*), two monocots (*Z. mays* and *O. sativa*), and seven dicots (*Arabidopsis*, *S. lycopersicum*, *C. sativus*, *P. trichocarpa*, *B. campestris*, *B. rapa*, and *B. napus*) (Figure 3). Intriguingly, *C. reinhardtii* possessed a solitary DOF member, which clustered into Subgroup D_1_. In terrestrial plants, moderate family sizes were observed in *O. sativa* (30), *Z. mays* (46), and *A. thaliana* (36). In comparison, 67, 74, and 117 DOF members were identified in *B. campestris*, *B. rapa*, and *B. napus*, respectively, indicating substantial expansion of the DOF family within *Brassica* species. Given its presence in the green alga *C. reinhardtii* and its broad conservation across land plants, Subgroup D_1_ may represent an ancient and highly conserved DOF lineage.

### 3.3. Orthologous Gene Clustering of DOF Families in Brassicaceae

**Figure 4:**
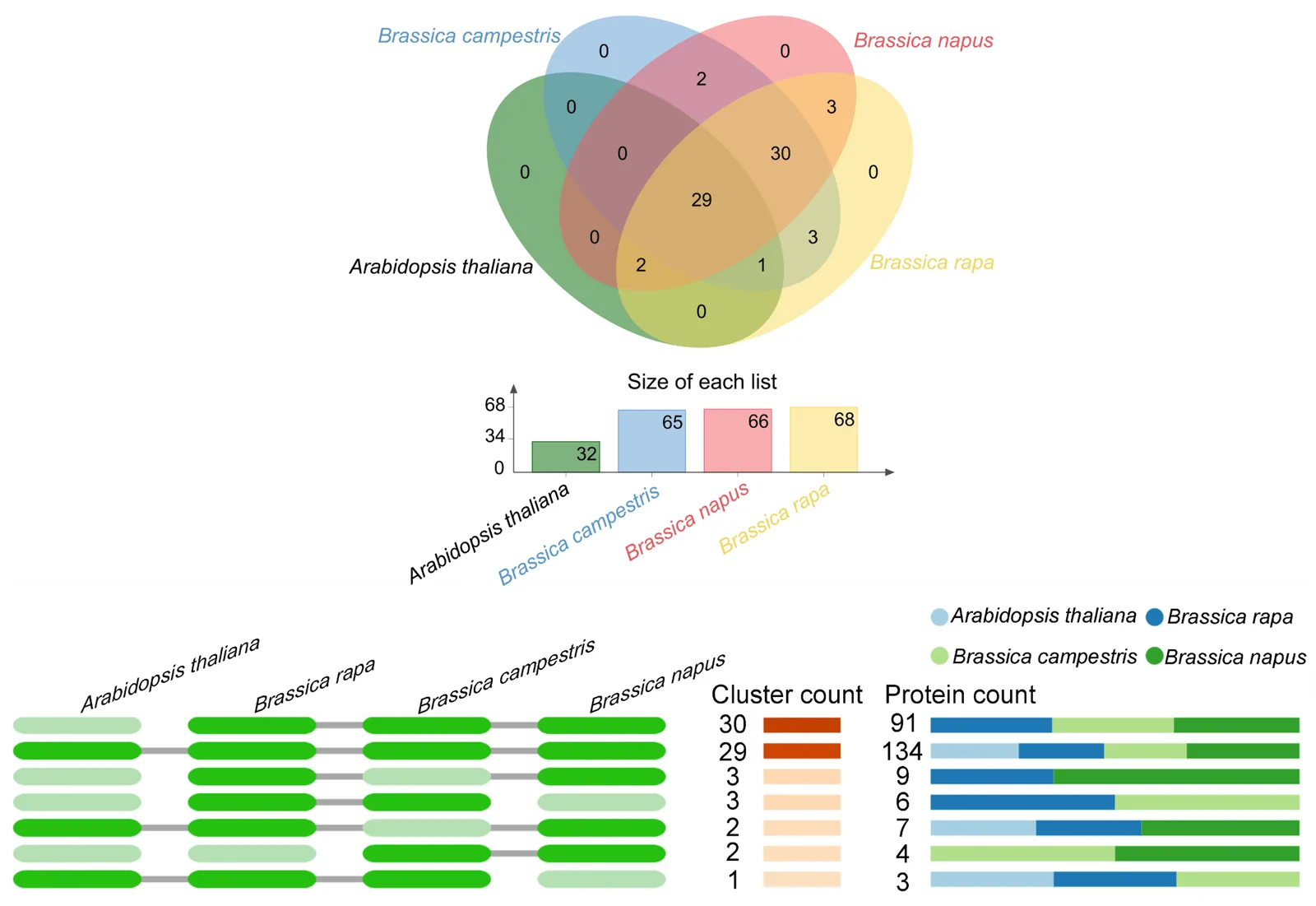
Orthologous clustering of DOF proteins among *B. campestris*, *B. rapa*, *B. napus*, and *Arabidopsis*. Orthologous groups were determined via the OrthoVenn3 platform with an E-value threshold of 1 × 10^−10^.

Given the pronounced expansion of DOF proteins observed in *Brassica* species, we performed an orthologous clustering analysis across four representative Brassicaceae species using the OrthoVenn3 platform, incorporating 294 DOF proteins from *B. campestris* (67), *B. rapa* (74), *B. napus* (117), and *A. thaliana* (36) (Figure 4). Among the 70 orthologous clusters, 29 clusters comprising 134 DOF proteins were shared across all four species, whereas 30 clusters encompassing 91 DOFs were identified as *Brassica*-specific. Pairwise comparisons revealed three shared clusters (6 DOFs) between *B. campestris* and *B. rapa*, three (9 DOFs) between *B. rapa* and *B. napus*, and two (4 DOFs) between *B. campestris* and *B. napus*. Additionally, three-species overlaps were detected, with two clusters (7 DOFs) shared among *Arabidopsis*, *B. rapa*, and *B. napus*, and one cluster (3 DOFs) common to *Arabidopsis*, *B. campestris*, and *B. rapa*. In addition, 40 proteins were assigned as singletons, including one gene each from *Arabidopsis* (AT3G45610.1) and *B. campestris* (BcDOF14), alongside four from *B. rapa*, and 34 from *B. napus*. Detailed information regarding these orthologous clusters and singletons is listed in Tables S2 and S3.

### 3.4. Conserved Motifs and Gene Structural Features of BcDOFs

**Figure 5:**
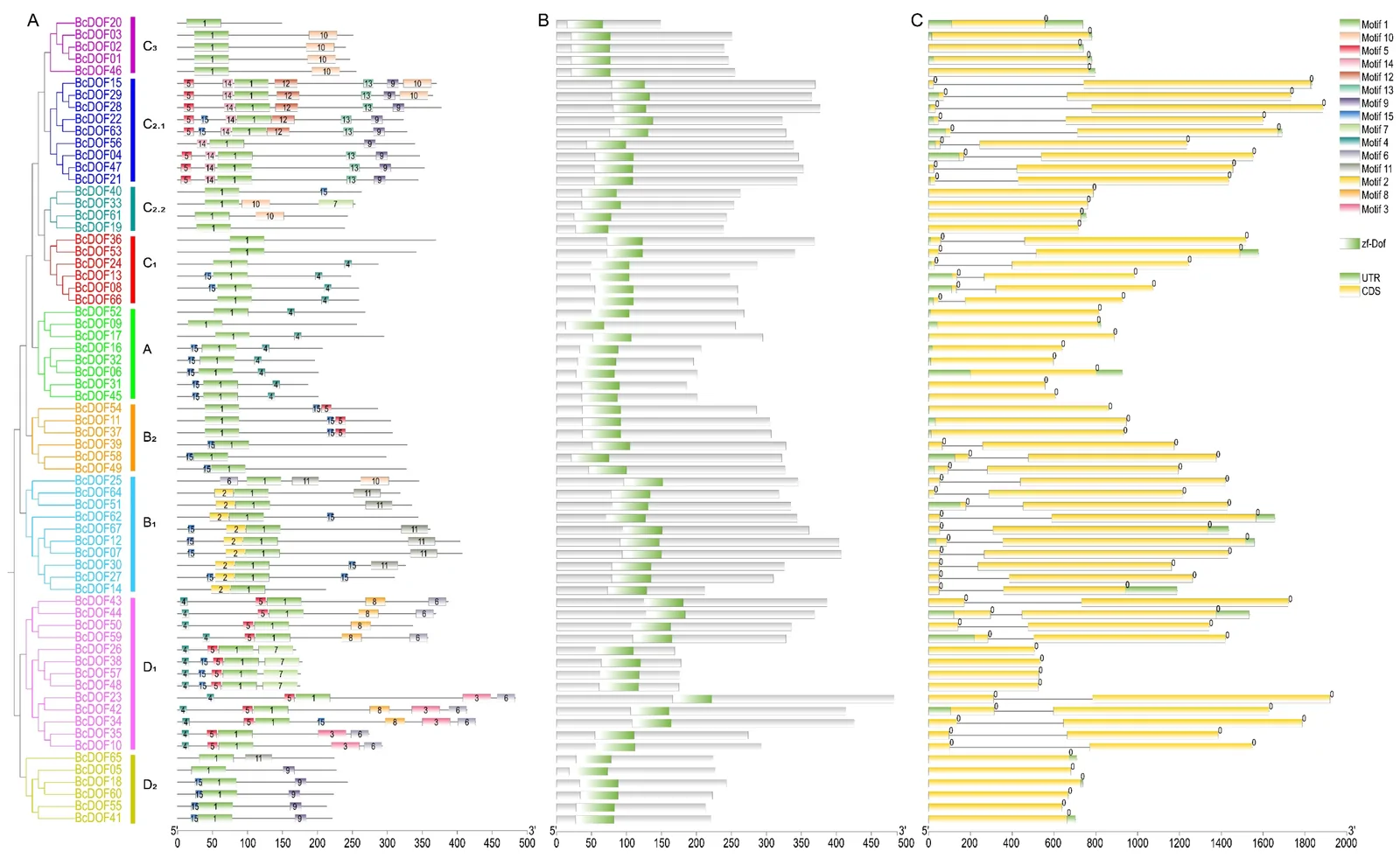
Comprehensive phylogenetic, motif, domain, and gene structural profiling of BcDOFs. (**A**) Phylogenetic tree and conserved motif distribution. Numbered colored boxes represent different motifs; (**B**) conserved domain architecture. Green boxes indicate the characteristic zinc-finger DOF (zf-DOF) domain; (**C**) exon–intron patterns. Yellow rectangles, green rectangles, and black lines represent UTRs, exons, and introns, respectively. Numbers above the lines indicate intron phases.

To delineate the structural architectures of BcDOFs, conserved motifs and gene structures were systematically analyzed. In total, 15 distinct motifs were detected across 67 BcDOFs using MEME Suite (Figure 5A,B; Figure S1). Notably, Motif 1, corresponding to the signature zf-DOF domain, was present in all BcDOF proteins. In contrast, the remaining motifs exhibited clade-specific distribution patterns, primarily within the C-terminal regions. For instance, Subgroups C_2.2_ and C_3_ were characterized by Motif 10, whereas Subgroup C_2.1_ carried N-terminal Motifs 5/14 along with C-terminal Motifs 9/10/12. Subgroups A and C_1_ shared Motifs 4 and 15, while Subgroup D_1_ exhibited the greatest motif diversity, harboring Motifs 3–8 and 15 (Figure 5A).

Analysis of exon–intron architecture revealed pronounced divergence among *BcDOF* subgroups (Figure 5C). Members of Subgroups A, C_2.2_, C_3_, and D_2_ were universally intronless, whereas Subgroups C_1_, C_2.1_, and B_1_, alongside the majority of B_2_ and D_1_ members, possessed a single intron. Such structural divergence implies that gene structural reorganization may play a pivotal role in functional specialization during *BcDOF* evolution.

### 3.5. Collinearity and Duplication Analysis of BcDOF Genes

**Figure 6:**
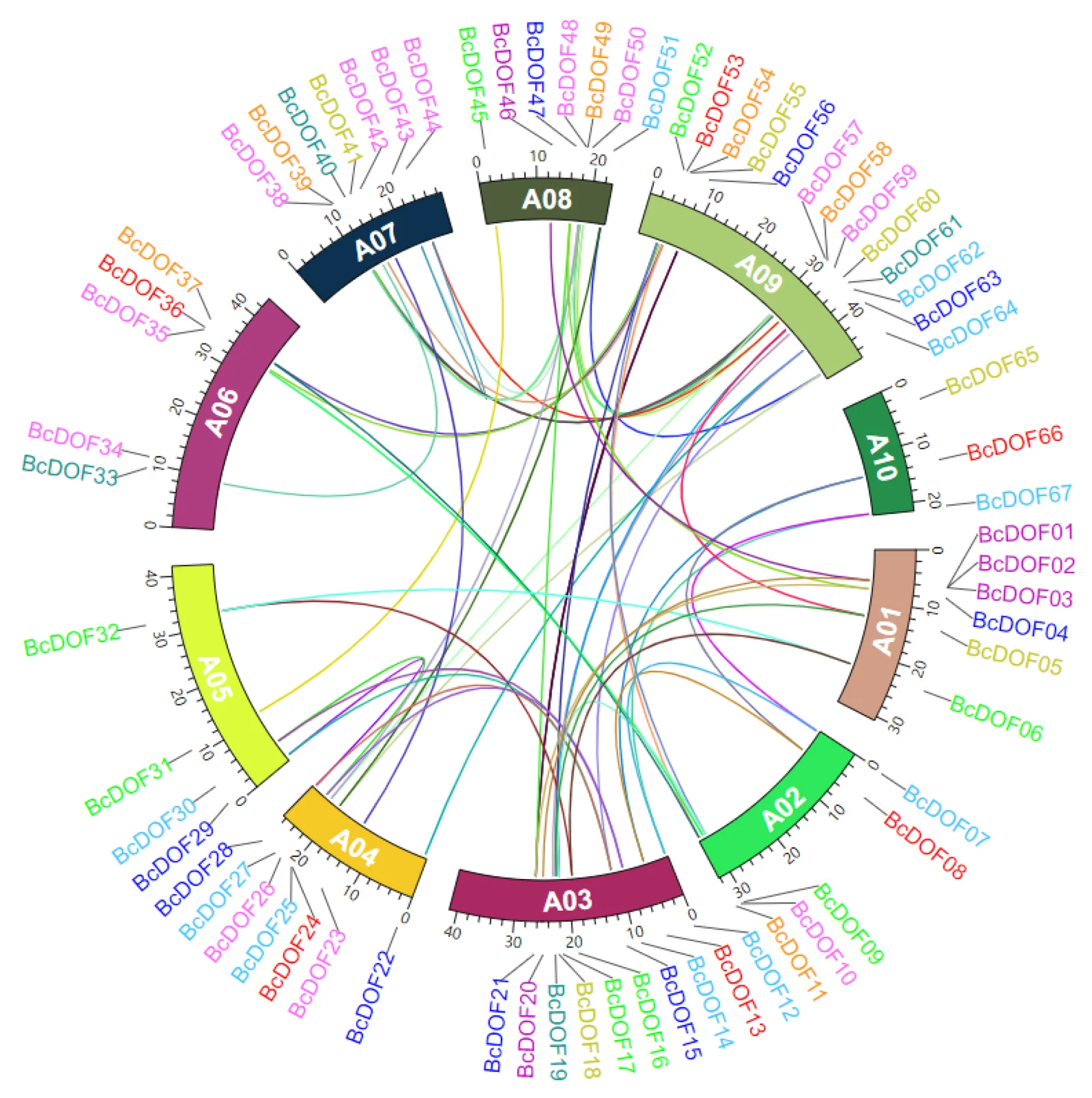
Collinearity relationships of *DOF* genes within *B. campestris*. Colored boxes represent the chromosomes of *B. campestris*, and colored connecting lines denote paralogous gene pairs.

To elucidate the evolutionary history of *DOF* genes, both intraspecific collinearity analysis of *B. campestris* and interspecific synteny analysis across *B. campestris*, *B. rapa*, *B. napus*, and *Arabidopsis* were performed using TBtools-II v.2.482 (Figure 6; Table S4). Among the identified *BcDOF* genes, two gene pairs were classified as tandem duplicates (*BcDOF01*/*BcDOF02* and *BcDOF02*/*BcDOF03*), four genes (*BcDOF24*, *BcDOF34*, *BcDOF62*, and *BcDOF65*) were classified as products of dispersed duplication, while the majority arose from whole-genome triplication (WGT) or segmental duplication. Consequently, WGT or segmental duplication predominantly drove the expansion of *BcDOF* genes in *B. campestris*.

To assess the selective constraints and evolutionary rates acting on duplicated *BcDOF* genes, we calculated the nonsynonymous-to-synonymous substitution ratios (Ka/Ks) across 56 paralogous pairs (Table S5). The estimated divergence times ranged from 3.84 (*BcDOF02* and *BcDOF03*) to 47.28 (*BcDOF25* and *BcDOF51*) million years ago (MYA), averaging 12.07 MYA. Notably, one gene pair (*BcDOF21* and *BcDOF56*) was excluded from subsequent analysis due to extremely high sequence divergence. Only one gene pair (*BcDOF01*/*BcDOF02*) exhibited neutral selection (Ka/Ks ≈ 1), whereas the remaining pairs had Ka/Ks ratios varying from 0.15 (*BcDOF14*/*BcDOF30*) to 0.82 (*BcDOF02*/*BcDOF03*). These findings suggest that purifying selection has dominated *BcDOF* evolution, thereby preserving the structural integrity and functional conservation of the *BcDOF* family during long-term evolution.

**Figure 7:**
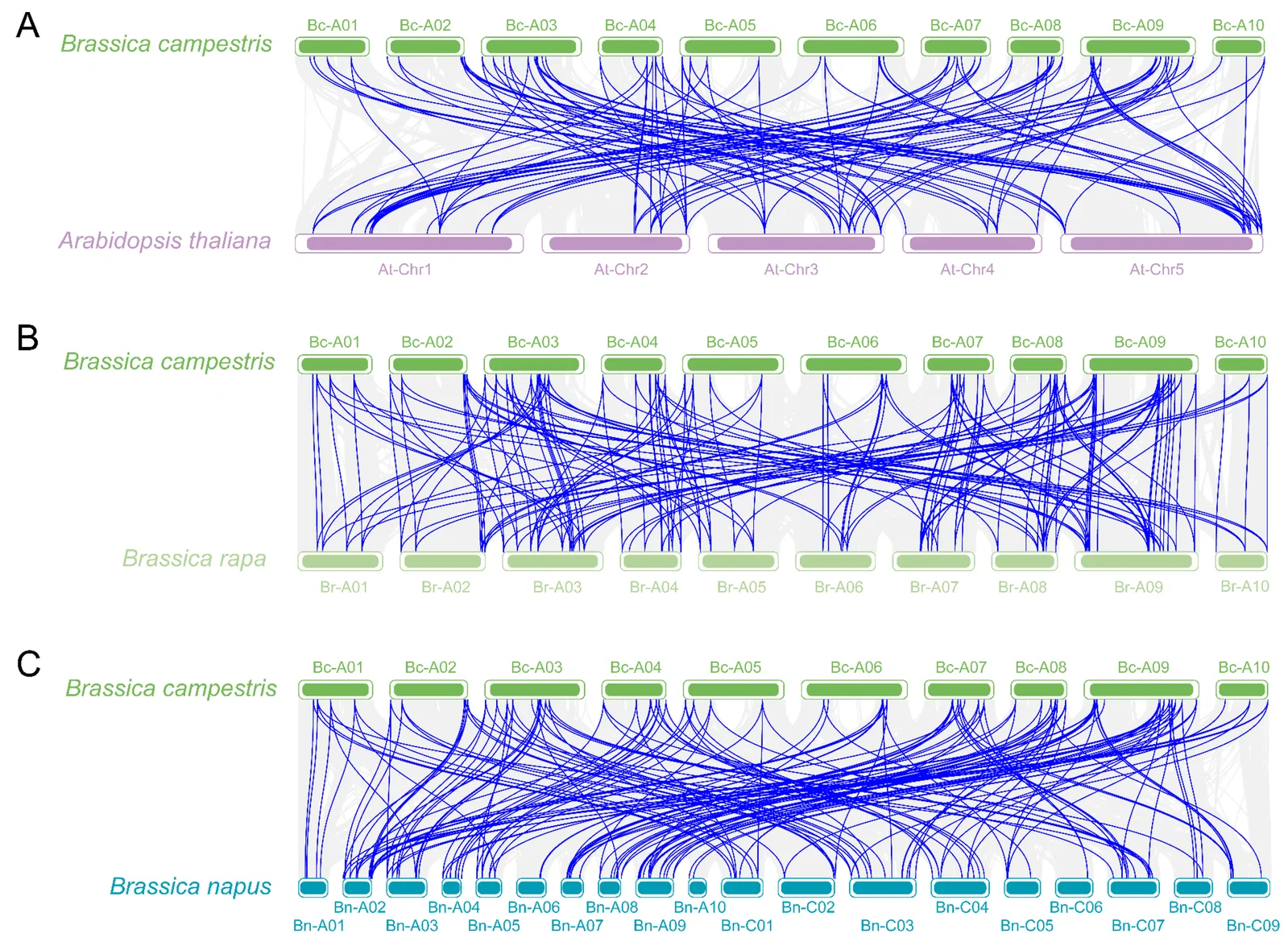
Synteny analysis of *DOF* genes across *B. campestris* and other plant species. (**A**) *B. campestris* and *A. thaliana*; (**B**) *B. campestris* and *B. rapa*; (**C**) *B. campestris* and *B. napus*. Chromosomes of each species are represented by distinct colored rectangles. Gray links in the background denote macro-syntenic blocks between genomes, whereas blue lines highlight conserved *DOF* syntenic gene pairs.

Interspecific synteny analysis identified 104, 162, and 165 homologous gene pairs between *B. campestris* and *A. thaliana*, *B. rapa*, and *B. napus*, respectively (Figure 7; Table S6). The Ks values of these homologous pairs between *B. campestris* and *Arabidopsis* ranged from 0.25 to 2.88, with an average of 0.69, corresponding to an average divergence time of approximately 23.13 MYA (Table S6). For interspecific orthologous gene pairs within *Brassica* species, the Ks values varied from 0 to 1.43, corresponding to average divergence times of 3.86 to 6.81 MYA. These results indicate that, following its split from the common ancestor shared with *Arabidopsis*, the *Brassica* lineage experienced WGT, which reshaped the modern genomes and drove the expansion of the *DOF* family across *Brassica* species.

### 3.6. Analysis of cis-Acting Elements in BcDOF Promoters

**Figure 8:**
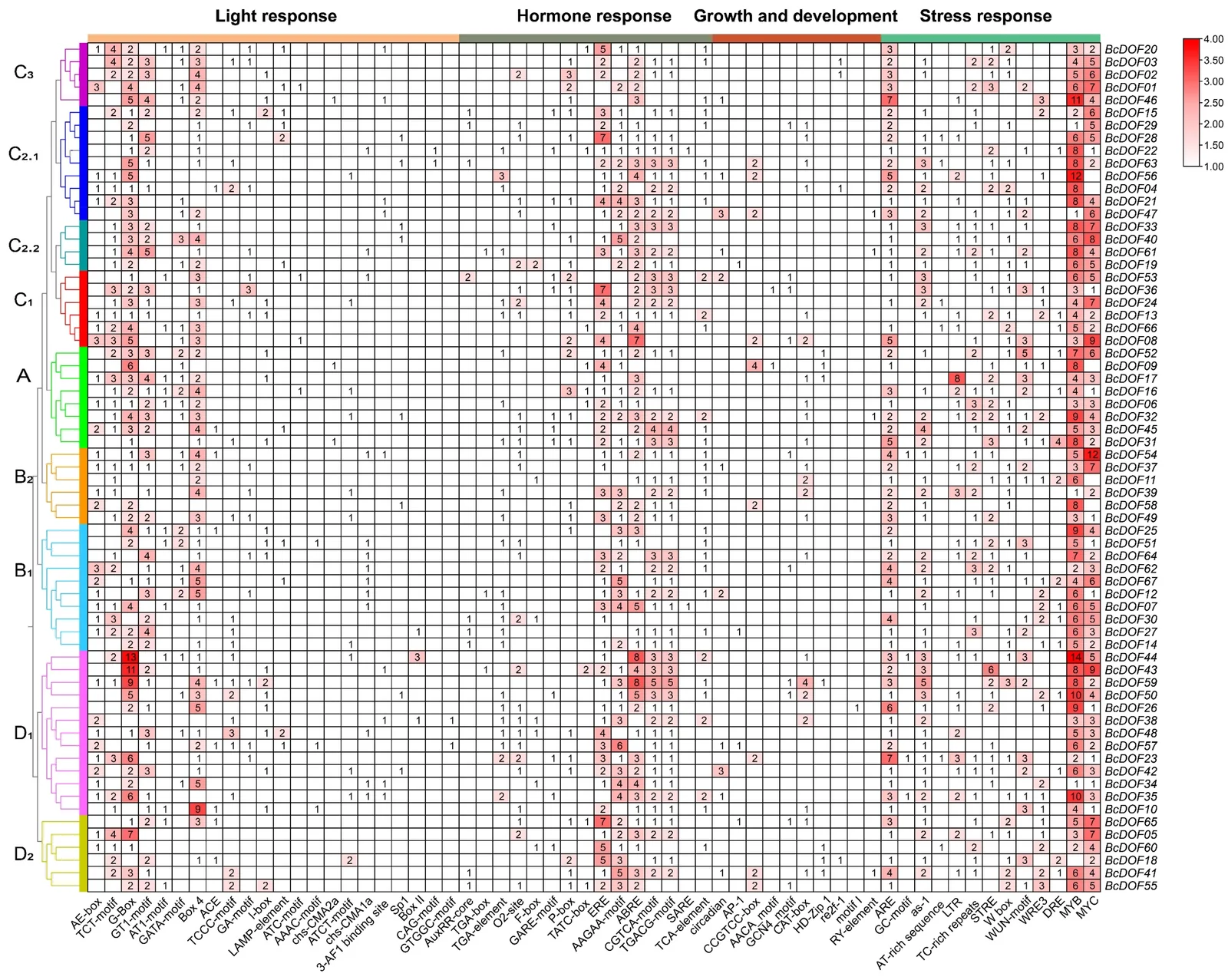
*Cis*-acting element analysis of *BcDOF* promoters. The ML phylogenetic tree was constructed with MEGA 7.0. Colored bars on the left represent BcDOF subfamilies, whereas horizontal bars at the top indicate functional categories. Numbers within each box represent the number of corresponding elements in *BcDOF* promoters.

To elucidate the transcriptional regulatory mechanisms governing *BcDOFs*, we analyzed the 2000 bp promoter regions using the PlantCARE database. In total, 60 distinct *cis*-acting elements were mapped and assigned to four functional modules: light response, phytohormone signaling, growth and development, and environmental stress response (Figure 8; Table S7).

Among all functional classes, elements responsive to light predominated in both frequency and diversity, spanning 22 distinct motif types. The G-box, Box-4, GT1-motif, and TCT-motif were the most prevalent, occurring in 85.07%, 82.09%, 65.67%, and 59.70% of *BcDOF* promoters, respectively. Phytohormone-responsive elements constituted another major category, encompassing elements associated with auxin (AuxRR-core, TGA-box, and TGA-element), gibberellin (TATC-box, P-box, and GARE-motif), abscisic acid (ABRE and AAGAA-motif), ethylene (ERE), methyl jasmonate (TGACG- and CGTCA-motif), and salicylic acid (SARE and TCA-element). Notably, ABRE elements were the most prevalent, occurring 137 times in 55 *BcDOF* promoters (82.09%). In terms of developmental regulation, several organ-specific elements were recognized, including those modulating endosperm metabolism (GCN4-motif and AACA-motif), root-specific expression (motif I), meristem differentiation (CAT-box and CCGTCC-box), flower development (AP-1), and seed maturation (RY-element). Furthermore, 13 stress-responsive elements were detected, dominated by anaerobic-induction elements (ARE, present in 92.54% of promoters with 142 sites), followed by pathogen defense signaling (as-1, 76.12%), wounding (WUN-motif, 46.27%), and low temperature (LTR, 41.79%), alongside other defense elements (STRE, W-box, TC-rich repeats, and WRE3). Abundant MYB and MYC transcription factor binding sites were also detected across *BcDOF* promoters. Taken together, the extensive *cis*-acting element profiles suggest that *BcDOFs* act as pivotal regulatory nodes integrating phytohormonal signaling and environmental stimuli to coordinate development and stress adaptation in *B. campestris*.

### 3.7. Organ-Specific Expression Profiles of BcDOF Genes

**Figure 9:**
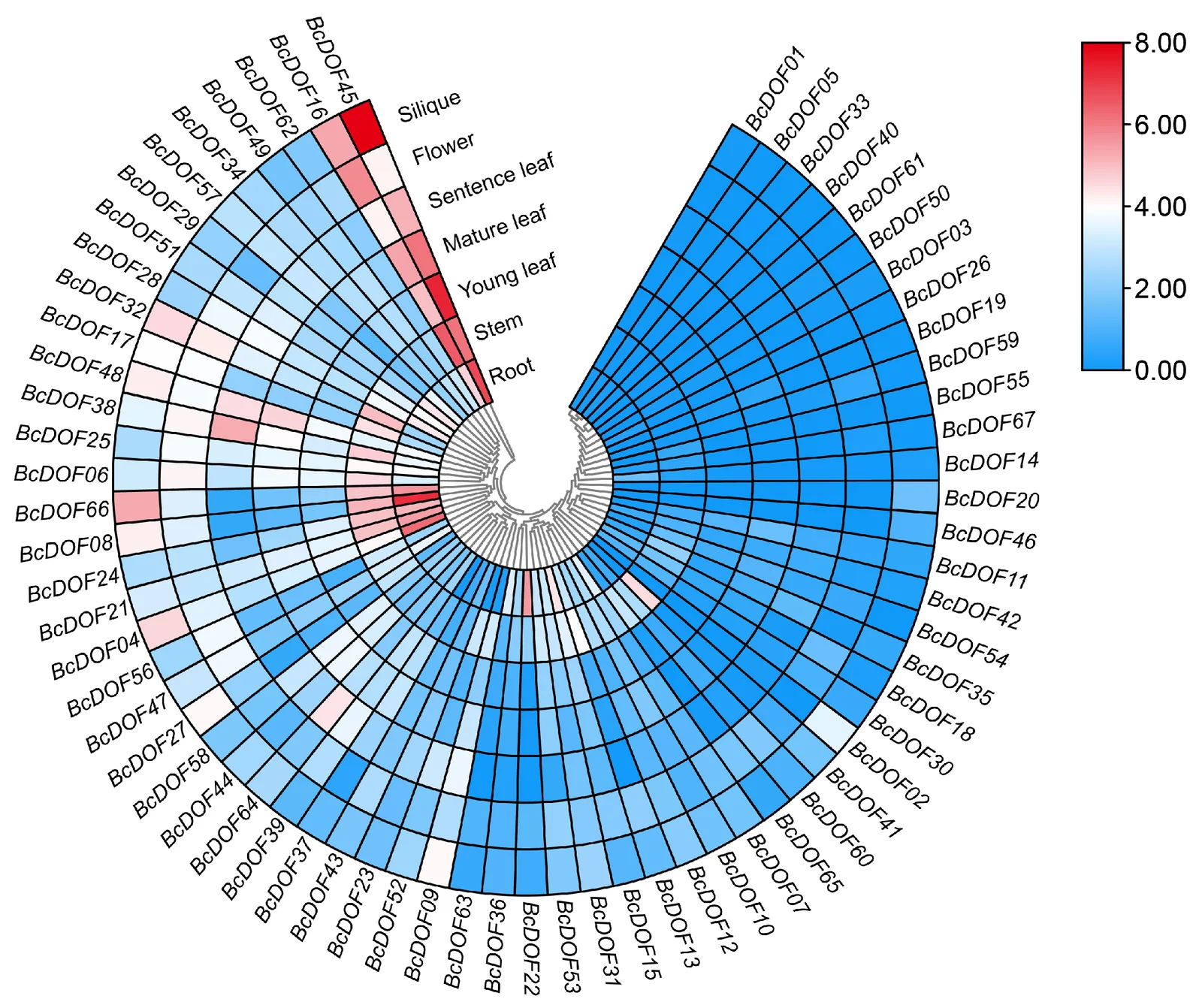
Organ expression profiles of *BcDOFs* in *B. campestris*. RNA-seq data were acquired from roots, stems, flowers, siliques, young leaves, mature leaves and senescent leaves of *B. campestris*.

To investigate the spatial expression profiles of *BcDOFs*, publicly available RNA-seq data (SRP427920) of *B. campestris* cultivar ‘49 Caixin’ were analyzed across seven representative organs, including roots, stems, flowers, siliques, young leaves, mature leaves, and senescent leaves (Figure 9).

*BcDOF* genes exhibited highly divergent expression patterns across the surveyed organs. Five genes exhibited ubiquitous expression, with *BcDOF16* and *BcDOF45* constitutively expressed at high levels, whereas *BcDOF34*, *BcDOF49*, and *BcDOF62* demonstrated broad expression with no marked organ bias. By contrast, several genes showed pronounced spatial preference: *BcDOF28*, *BcDOF29*, *BcDOF51*, and *BcDOF57* were predominantly expressed in roots; *BcDOF17*, *BcDOF32*, *BcDOF38*, and *BcDOF48* were preferentially expressed in stems, flowers, and siliques; and a distinct cluster comprising *BcDOF04, BcDOF08*, *BcDOF21*, *BcDOF24*, and *BcDOF66* shared elevated transcript abundance in roots, stems, and siliques. Additionally, 22 *BcDOF* members (e.g., *BcDOF01*, *BcDOF02*, *BcDOF05*, *BcDOF61*) displayed negligible or basal expression throughout all surveyed organs, implying stage-specific or conditional activation.

### 3.8. Expression Analysis of BcDOFs Under Abiotic Stress

To comprehensively characterize the stress-responsive profiles of *BcDOFs*, 12 representative family members were selected for RT-qPCR analysis. These candidates exhibit high basal transcript abundance in both leaves and roots and cover the majority of chromosomal loci. Five-leaf-stage *B. campestris* seedlings were subjected to heat stress (40 °C) and PEG-induced osmotic stress (10% PEG 6000), and the relative transcript levels of 12 selected *BcDOF*s in roots and leaves were quantified.

**Figure 10:**
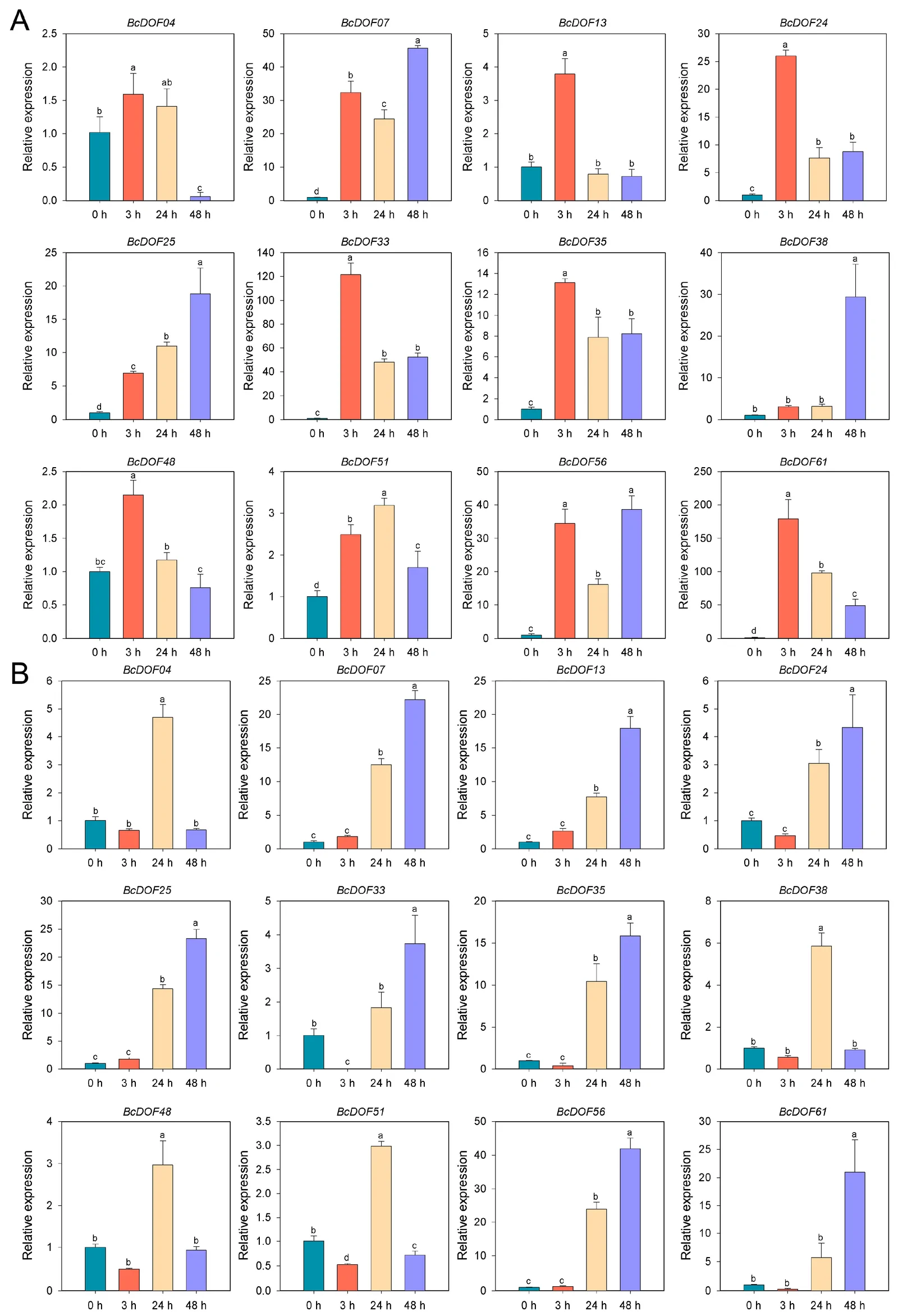
Expression profiles of *BcDOFs* under heat stress in *B. campestris*. Leaf (**A**) and root (**B**) profiles are illustrated. The horizontal and vertical axes depict treatment time and relative expression levels, respectively. Values are expressed as mean ± standard deviation (SD, *n* = 3). Treatment differences were analyzed via one-way ANOVA followed by Tukey’s Honestly Significant Difference (HSD) test, and different lowercase letters denote significant differences at *p* < 0.05.

Under heat stress, the majority of *BcDOF* genes in leaves were significantly induced at 3 h after treatment. Transcripts of *BcDOF13*, *BcDOF24*, *BcDOF33*, *BcDOF35*, *BcDOF48*, and *BcDOF61* peaked at 3 h and subsequently declined at 24 and 48 h, yet remained significantly elevated relative to the control. *BcDOF07*, *BcDOF25*, and *BcDOF56* exhibited continuous increases during the treatment period, reaching maximum expression at 48 h. In contrast, *BcDOF04* was transiently induced at the early stage before being sharply repressed by 48 h (Figure 10A). In roots, most *BcDOFs* displayed slight changes at 3 h but were markedly upregulated at later stages. *BcDOF04*, *BcDOF38*, *BcDOF48*, and *BcDOF51* exhibited strong induction at 24 h, whereas the remaining tested genes maintained elevated transcript abundance at both 24 and 48 h (Figure 10B).

**Figure 11:**
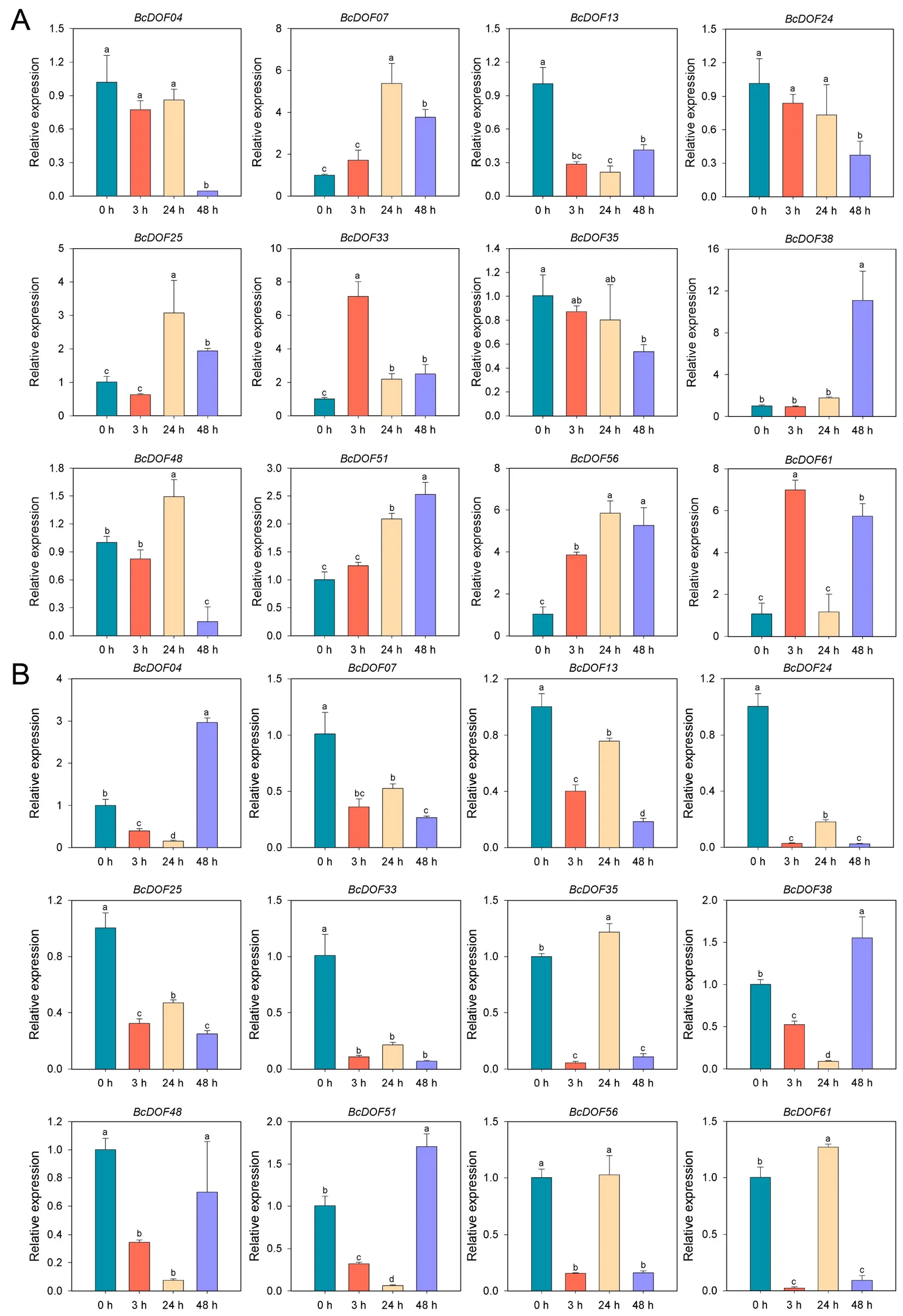
Expression profiles of *BcDOFs* under osmotic stress in *B. campestris*. Leaf (**A**) and root (**B**) profiles are illustrated. The horizontal and vertical axes depict treatment time and relative expression levels, respectively. Values are expressed as mean ± SD (*n* = 3). Treatment differences were analyzed via one-way ANOVA followed by Tukey’s HSD test, and different lowercase letters denote significant differences at *p* < 0.05.

PEG-induced osmotic stress elicited divergent expression responses among *BcDOFs*. In leaves, most *BcDOFs* were significantly induced by osmotic stress. *BcDOF33* and *BcDOF61* responded rapidly, peaking at 3 h; *BcDOF07*, *BcDOF25*, *BcDOF48*, and *BcDOF56* reached maximum transcript abundance at 24 h, whereas *BcDOF38* and *BcDOF51* peaked at 48 h (Figure 11A). Conversely, *BcDOF04*, *BcDOF13*, *BcDOF24*, and *BcDOF35* were significantly downregulated throughout the treatment. In roots, the majority of *BcDOFs* were initially suppressed at the early stage of osmotic stress but recovered at subsequent time points. Notably, *BcDOF04*, *BcDOF38*, and *BcDOF51* were markedly upregulated at 48 h relative to the control (Figure 11B). Collectively, these data indicate that *BcDOFs* display distinct transcriptional dynamics under heat and osmotic stresses, supporting their functional diversification in conferring abiotic stress resilience in *B. campestris*.

Relative to other *BcDOF* family members, *BcDOF56* exhibits sustained and marked stress-induced upregulation, with its promoter harboring abundant stress- and hormone-responsive *cis*-acting elements. Therefore, *BcDOF56* was selected as the preferred candidate for further functional characterization.

### 3.9. Subcellular Localization of BcDOF56

**Figure 12:**
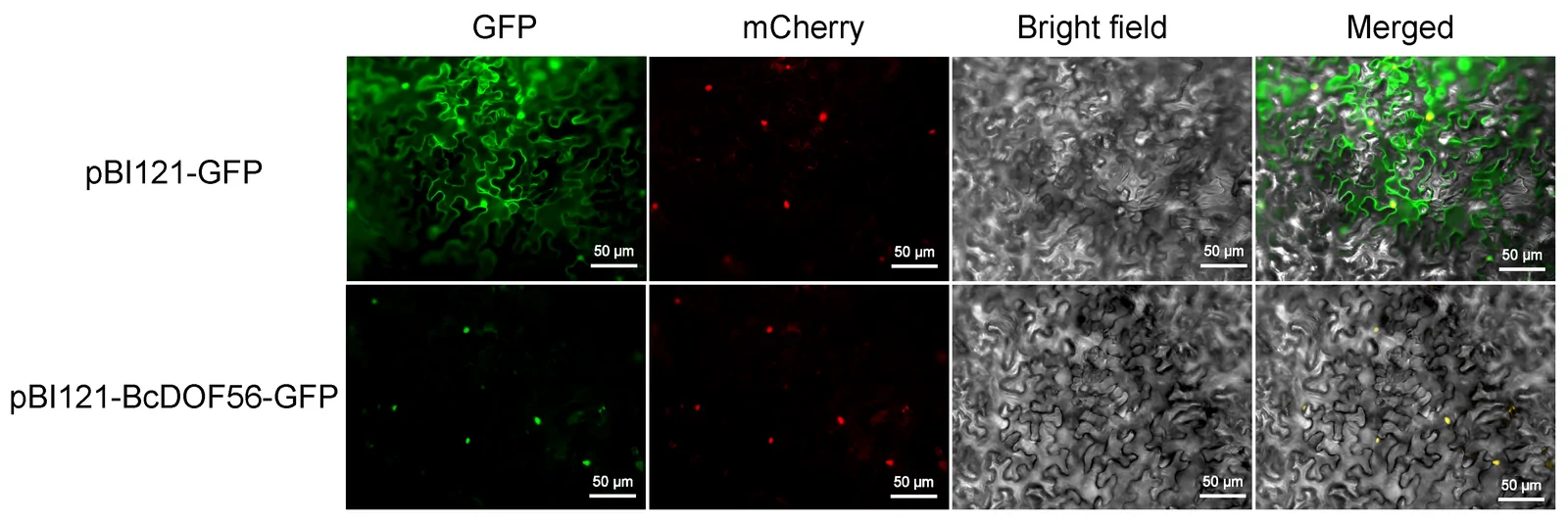
Subcellular localization of BcDOF56 protein. BcDOF56 was fused with GFP, and nuclear localization signal (NLS)-mCherry was used as a nuclear marker to visualize its subcellular localization. Scale bar = 50 μm.

To determine the subcellular distribution of BcDOF56, the fusion construct pBI121-BcDOF56-GFP was transiently expressed in *N. benthamiana* leaves. The GFP fluorescence strictly co-localized with the nuclear marker NLS-mCherry (Figure 12), verifying that BcDOF56 resides in the nucleus, in line with its functional annotation as a transcriptional regulator.

### 3.10. Overexpression of BcDOF56 Enhances Heat and Drought Tolerance in Arabidopsis

**Figure 13:**
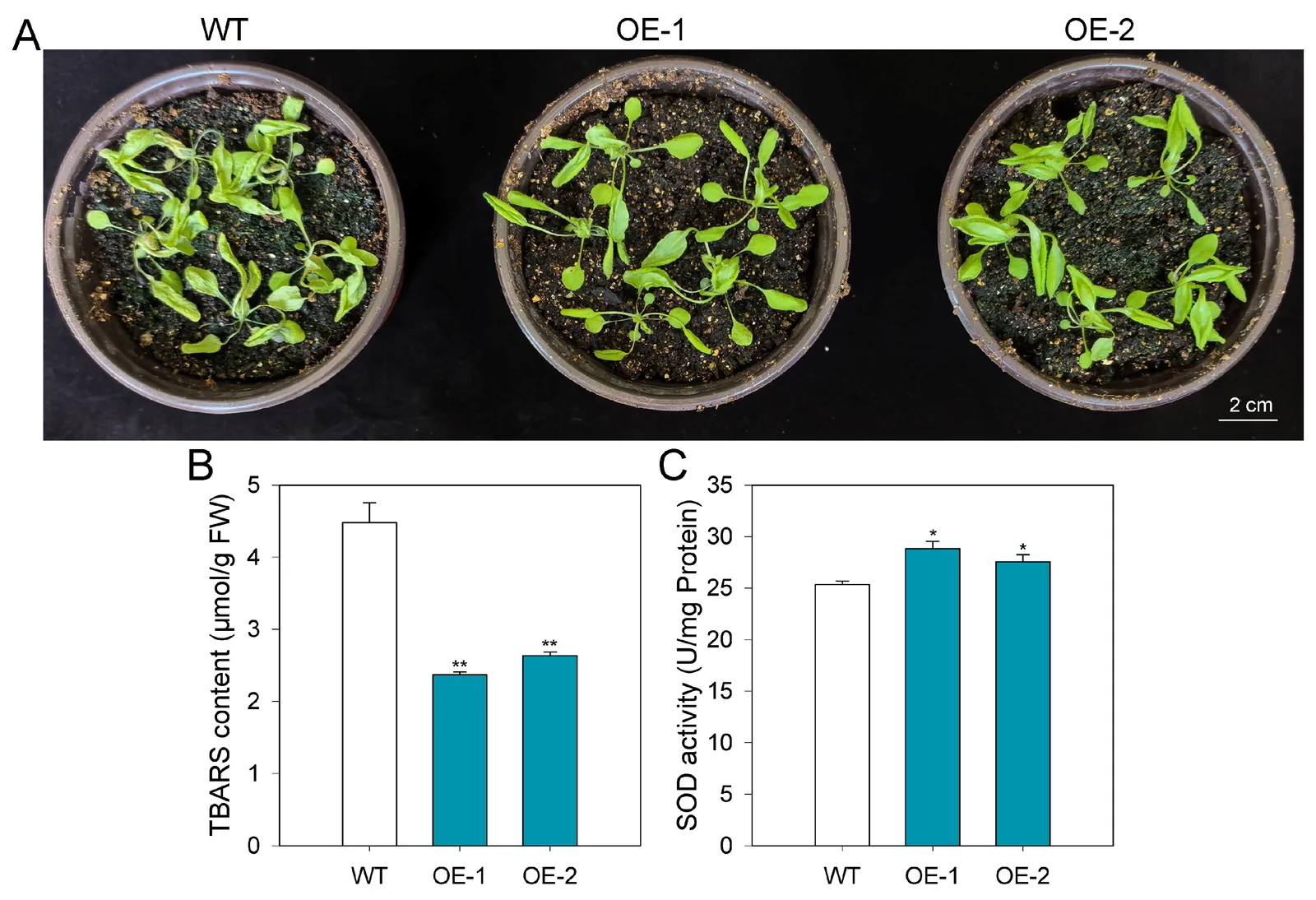
Functional characterization of *BcDOF56-*overexpressing *Arabidopsis* under heat stress. (**A**) Growth phenotypes of wild-type (WT) and *BcDOF56*-OE lines (OE-1 and OE-2) exposed to 40 °C for 60 h; (**B**) thiobarbituric acid reactive substances (TBARS) content of leaves in WT and transgenic lines; (**C**) superoxide dismutase (SOD) activity in leaves. All data are presented as mean ± SD (*n* = 3). Significance between WT and transgenic plants was evaluated via Student’s *t*-test (* *p* < 0.05 and ** *p* < 0.01).

To evaluate the biological functions of *BcDOFs* under environmental stress, *BcDOF56* was chosen for transgenic functional validation. Two independent T_3_ homozygous *Arabidopsis* lines overexpressing *BcDOF56* (Figure S2) were generated for stress tolerance assays. When four-week-old seedlings were exposed to 40 °C for 60 h, WT plants displayed severe leaf chlorosis and wilting; by contrast, the transgenic lines exhibited only mild leaf curling and slight wilting (Figure 13A). Consistent with these phenotypes, TBARS contents in transgenic lines were reduced to 52.94% and 58.84% of WT levels, while SOD activity was significantly elevated (Figure 13B,C).

**Figure 14:**
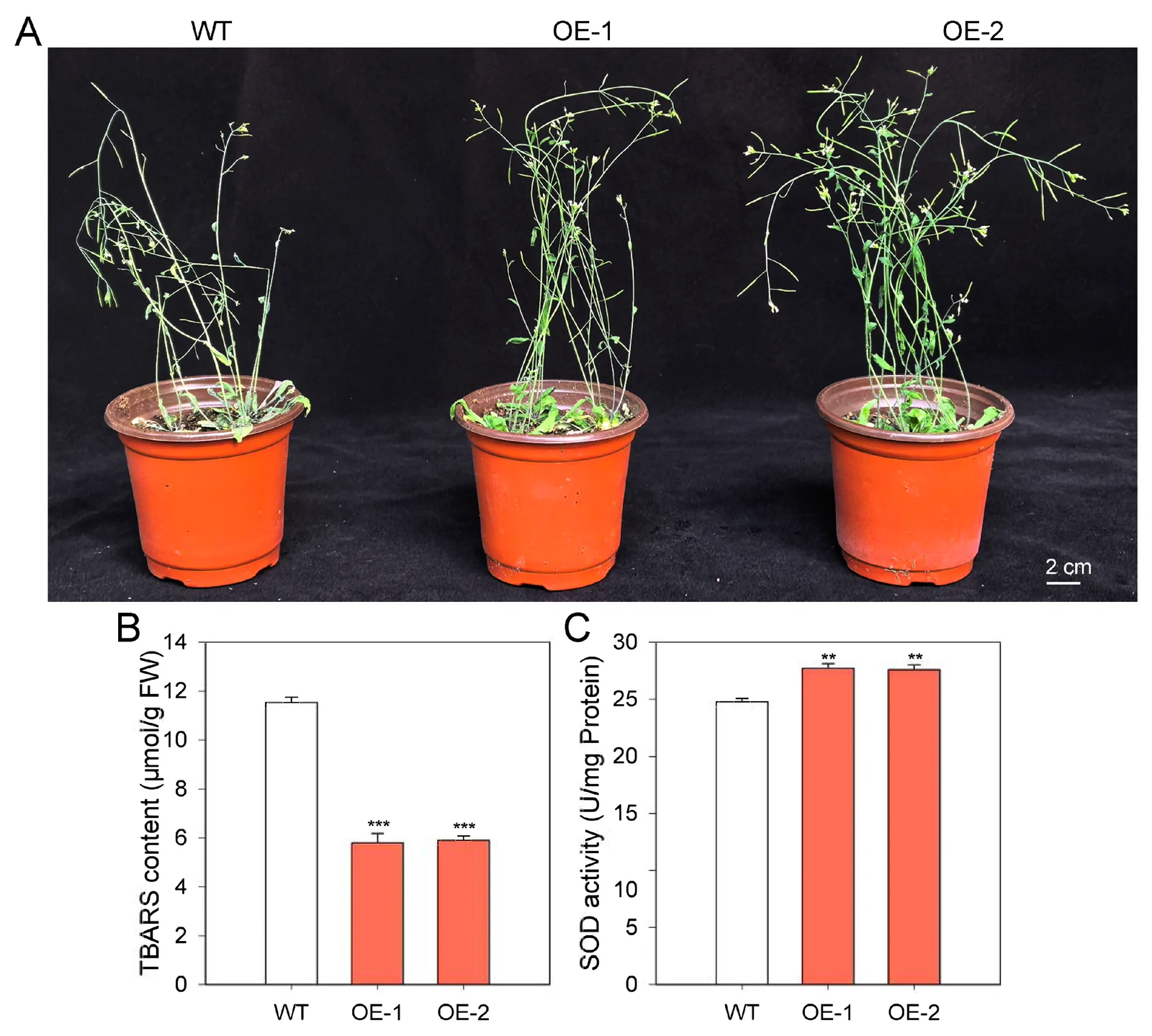
Functional characterization of *BcDOF56-*overexpressing *Arabidopsis* under drought stress. (**A**) Growth phenotypes of WT and *BcDOF56*-OE lines (OE-1 and OE-2) after 15 days of water deprivation; (**B**) TBARS content of leaves in WT and transgenic lines; (**C**) SOD activity in leaves. All data are presented as mean ± SD (*n* = 3). Significance between WT and transgenic plants was evaluated via Student’s *t*-test (** *p* < 0.01 and *** *p* < 0.001).

For drought treatment, water was withheld for 15 days, causing severe wilting and foliar chlorosis in WT plants, whereas transgenic lines maintained relatively healthy growth with only slight curling at the leaf margins (Figure 14A). Consistent with the observation under heat stress, transgenic lines accumulated lower TBARS (50.24% and 51.11% of WT) and exhibited elevated SOD activity relative to WT (Figure 14B,C). Collectively, these phenotypic and physiological data support a positive role for BcDOF56 in enhancing heat and drought tolerance in *Arabidopsis*.

## 4. Discussion

Plant-specific DOF factors function as pivotal hubs mediating development and responses to environmental stresses [4,23,38]. Although *DOF* gene families have been characterized in multiple plant species [12,13,14,15,16,17,26], a systematic characterization of the *DOF* family remains lacking in *B. campestris*. Therefore, we carried out a genome-wide identification of this family in *B. campestris*, followed by analyses of its evolutionary relationships, structural characteristics, expression profiling, and potential roles in heat and drought responses.

### 4.1. Evolutionary Dynamics and Expansion of the BcDOF Gene Family

In *B. campestris*, a total of 67 *BcDOF* genes were systematically identified. A comparative cross-species survey revealed a progressive expansion of *DOF* members from algae to angiosperms. *C. reinhardtii* harbors only a single *DOF* gene, whereas both monocot and dicot genomes possess substantially more members. This observation supports the hypothesis that the *DOF* gene family may have originated from a shared ancestor gene retained as a single copy in *C. reinhardtii* [15]. Throughout land plant evolution, the expansion of *DOF* genes was closely associated with the establishment of complex transcriptional networks that facilitate adaptation to fluctuating environments [4]. Within Brassicaceae, *Arabidopsis* harbors 36 *DOF* genes, whereas the three investigated *Brassica* species exhibit dramatic family expansion, especially *B. napus*, whose 117 members represent the largest *DOF* inventory documented to date [15]. The interspecific copy-number divergence is largely attributed to gene duplication events and modifications of genome size and structure [39].

Synteny analysis revealed that an average Ks value of 0.69 for orthologous *DOF* pairs between *Arabidopsis* and *B. campestris*, corresponding to a divergence time of approximately 23.13 MYA, is consistent with the published 14–24 MYA divergence time for *Arabidopsis* and *Brassica* species [15,40]. Following divergence from *Arabidopsis*, *Brassica* species underwent whole-genome triplication (WGT), chromosomal rearrangements, and allopolyploidization, which collectively reshaped their modern genomic architecture and catalyzed substantial gene family expansion [41]. Selection pressure analysis demonstrated that nearly all *BcDOF* paralog pairs exhibited Ka/Ks ratios substantially below 1.0, with only one gene pair (*BcDOF01*/*BcDOF02*) displaying a ratio close to 1. These findings suggest that purifying selection acted as the primary evolutionary constraint preserving BcDOF functional stability, while WGT and segmental duplication served as the major drivers expanding the *BcDOF* family to adapt to fluctuating environments [15].

### 4.2. Phylogenetic Classification, Structural Conservation and cis-Regulatory Characteristics

According to the phylogenetic classification established in *Arabidopsis* [12], all 67 BcDOF proteins were classified into nine subfamilies. Subgroup D_1_ comprised the largest proportion of members (19.40%), whereas Subgroup C_2.2_ contained the fewest (5.97%). This distribution pattern is consistent with that of *Arabidopsis* [12] and *B. rapa* [14], but departs from that reported in *B. napus* [15], potentially reflecting distinct evolutionary histories and functional diversification among lineages. The conserved zf-DOF domain was detected in all BcDOF proteins. DOFs within the same clade shared similar motif architectures, with Subgroup D_1_ exhibiting more motif types than other subgroups. This observation aligns with the findings in *B. rapa* [14], *B. napus* [15], and tobacco [17]. Furthermore, exon–intron architecture analysis revealed that *BcDOFs* possessed one or two exons, with the majority (55.22%) containing two exons, matching the structural features of the *DOF* family reported in *Arabidopsis* [12], *B. napus* [15], and tobacco [17].

*Cis*-acting element analysis revealed that *BcDOF* promoters harbor abundant elements responsive to abiotic stress (drought, low temperature, anaerobic induction) and phytohormones (ABA, SA, and MeJA). These features align closely with findings in *B. napus* [15], tobacco [17], and pea [42], further supporting the evolutionarily conserved role of DOF proteins in regulating abiotic stress tolerance [38,43]. Analogous promoter compositions have also been reported in kiwifruit *AcDOFs* and *Dendrobium officinale DoDOFs* [44,45]. The conservation of these *cis*-elements reinforces the notion that DOF transcription factors act as central regulatory hubs integrating phytohormone cascades with environmental stress adaptations [5,43,46].

### 4.3. Divergent Expression Dynamics of BcDOFs Under Heat and Drought Stresses

Previous studies have illustrated the vital functions of DOF proteins in plant responses and adaptation to environmental stress [38]. In *Arabidopsis*, AtDOF5.8 is significantly upregulated by drought and salt stress and enhances seedling stress resilience via interaction with ANAC069 [24]. In *B. napus*, most *BnaDOF* genes respond strongly to extreme temperatures; *BnaDOF54* (*BnCDF1*) is induced by cold [15], and its overexpression markedly improves cold tolerance [22]. Stress-responsive *DOF* homologs have also been implicated in stress responses in non-model species: *PeDOF-11* is strongly activated by heat stress in *Passiflora edulis* [47]; *DoDOF7* and *DoDOF26* are significantly upregulated by heat and drought in *Dendrobium officinale* [45]; and *PsDOFs* show broad stress-responsive expression across various tissues in *Populus simonii* [27].

In the present study, RT-qPCR revealed distinct stress-dependent expression profiles of *BcDOFs*. Most *BcDOFs* were significantly induced in both foliar and root tissues under heat stress (Figure 10). Under osmotic stress, *BcDOFs* exhibited distinct organ-specific expression patterns: several genes (*BcDOF07*, *BcDOF25*, *BcDOF33*, *BcDOF38*, *BcDOF51*, and *BcDOF56*) were markedly induced in leaves, whereas *BcDOF04* and *BcDOF13* were significantly repressed; furthermore, the majority of *BcDOFs* were significantly downregulated in roots (Figure 11). Similar heterogeneous and organ-specific *DOF* expression profiles have been reported in *B. rapa* [14] and *D. officinale* [45]. In tobacco, NtDOF40 and NtDOF63 even exert opposing regulatory effects on cold tolerance [17]. *DOF* genes exhibit divergent expression profiles and biological functions under diverse stresses, with these differences shaped by plant species, genotype, and even tissue type [15]. Collectively, these findings suggest that *DOF* paralogs may have undergone functional diversification. Different members may regulate distinct downstream stress-responsive genes, leading to variation in stress-tolerance phenotypes across species and tissues.

### 4.4. BcDOF56 Positively Regulates Stress Tolerance Through ROS Homeostasis

Given its marked upregulation at the transcript level under both heat and osmotic stresses, especially in leaves, *BcDOF56* was selected as a promising candidate for heterologous overexpression in *Arabidopsis*. Phenotypic and physiological analyses revealed that *BcDOF56*-overexpressing lines survived better than WT under both heat and drought treatments, with substantially lower TBARS content and significantly higher SOD activity (Figure 13 and Figure 14). TBARS, measured via the thiobarbituric acid assay, is a widely used biomarker for lipid peroxidation in plant tissues, consisting of a range of TBA-reactive carbonyl compounds with MDA as the major component [48]. MDA serves as a reliable marker for cellular lipid peroxidation, whereas SOD acts as a core antioxidant enzyme that catalyzes the dismutation of superoxide radicals, thereby alleviating reactive oxygen species (ROS) damage [49]. Consequently, these physiological results suggest that BcDOF56 may enhance heat and drought tolerance, potentially by promoting antioxidant defenses to alleviate ROS-induced oxidative damage.

Similar ROS-modulating circuits mediated by DOF have been reported in diverse species. For instance, in tobacco, NtDOF40 and NtDOF63 antagonistically regulate cold tolerance by differentially modulating antioxidant enzyme activities and ROS homeostasis [17]. In sweetpotato, IbDOF2.1 interacts with ABA-binding factor 2 (IbABF2) to trigger stomatal closure and ROS scavenging under drought conditions [50]. In tea (*Camellia sinensis*), CsDOF transcription factors act as a central regulatory hub under combined heat–drought stress, integrating mitogen-activated protein kinase (MAPK) and heat shock factor (HSF) signaling with the antioxidant system to limit excessive ROS accumulation and maintain redox homeostasis [51]. These findings collectively suggest that DOF transcription factors participate in a multifaceted defense regulatory network by mediating protein–protein interactions, integrating hormonal signals, and activating the transcription of downstream antioxidant and protective genes [52]. Although ROS homeostasis appears central, studies of BcDOF56 homologs suggest additional mechanisms. In *Arabidopsis*, the closest homologs of BcDOF56, AtDOF1.8 (AT1G64620) and AtDOF4.6 (AT1G24060), are well-established regulators of vascular cell differentiation and are classified as VASCULAR-RELATED DOF (VDOF) [53]. AtDOF4.6 interacts with XYLEM NAC DOMAIN 1 (XND1) to transcriptionally modulate the aquaporin genes AtPIP2;5 and AtPIP2;6, thereby contributing to drought adaptation [54]. Given the functional conservation of DOF homologs across plant species, it is suggested that in addition to regulating ROS homeostasis, BcDOF56 may also contribute to stress tolerance by modulating vascular development and water transport efficiency. Notably, water status parameters were not assessed in the present study. Accordingly, the conclusion regarding BcDOF56-mediated stress tolerance remains preliminary. Future work will include systematic quantification of water status and soil drought assays to further elucidate the underlying mechanisms.

In conclusion, this study systematically characterizes the *DOF* gene family in *B. campestris* and identifies *BcDOF56* as a promising candidate gene for breeding climate-resilient crop varieties. However, the molecular mechanisms underlying BcDOF56-mediated heat and drought tolerance remain to be elucidated.

## 5. Conclusions

In the present study, we identified 67 *BcDOF* genes in flowering Chinese cabbage (*Brassica campestris*) and assigned them to nine subfamilies. All BcDOFs harbor a conserved zf-DOF domain, display distinct gene structures, and contain numerous *cis*-acting elements related to hormone signaling and stress responses. Phylogenomic analysis indicated that the DOF family has an ancient origin and underwent substantial expansion in angiosperms. In *B. campestris*, whole-genome triplication and segmental duplication were the primary drivers of the *BcDOF* family expansion. Expression profiling demonstrated that *BcDOFs* exhibit diverse responses to heat and drought stresses. Moreover, heterologous overexpression of *BcDOF56* in *Arabidopsis* enhanced tolerance to heat and drought stresses, possibly through the modulation of ROS homeostasis. Collectively, this work offers a framework for understanding the evolution and stress-responsive roles of the *BcDOF* family and identifies *BcDOF56* as a potential target gene for molecular breeding to improve abiotic stress tolerance.

## Figures and Tables

**Table 1:** Characteristics of BcDOF proteins in *B. campestris*.

Gene Name	Gene ID	Category	Number of Amino Acids	Molecular Weight/kDa	Theoretical pI	Instability Index	Aliphatic Index	Grand Average of Hydropathicity	Subcellular Localization
*BcDOF01*	Bra_cxA01g035470.1	C_3_	245	27.65	9.33	49.74	46.41	−0.835	Nucleus
*BcDOF02*	Bra_cxA01g035460.1	C_3_	239	27.23	9.18	40.96	51.67	−0.877	Nucleus
*BcDOF03*	Bra_cxA01g035450.1	C_3_	250	28.31	8.81	33.59	49.40	−0.86	Nucleus
*BcDOF04*	Bra_cxA01g032720.1	C_2.1_	346	37.51	7.69	48.55	46.50	−0.834	Nucleus
*BcDOF05*	Bra_cxA01g024090.1	D_2_	226	23.69	9.41	31.88	60.84	−0.323	Nucleus
*BcDOF06*	Bra_cxA01g013830.1	A	200	21.52	9.46	63.09	40.50	−0.973	Nucleus
*BcDOF07*	Bra_cxA02g049410.1	B_1_	406	42.81	9.26	60.60	53.65	−0.644	Nucleus
*BcDOF08*	Bra_cxA02g039610.1	C_1_	258	28.08	8.64	52.49	46.16	−0.744	Nucleus
*BcDOF09*	Bra_cxA02g003710.1	A	255	27.84	6.64	41.16	45.14	−0.779	Nucleus
*BcDOF10*	Bra_cxA02g002750.1	D_1_	292	33.37	8.71	42.01	56.37	−0.885	Nucleus
*BcDOF11*	Bra_cxA02g000600.1	B_2_	304	34.13	8.79	48.89	53.55	−0.863	Nucleus
*BcDOF12*	Bra_cxA03g066400.1	B_1_	403	42.48	9.17	62.05	51.84	−0.611	Nucleus
*BcDOF13*	Bra_cxA03g056590.1	C_1_	247	26.84	8.94	58.12	46.28	−0.687	Nucleus
*BcDOF14*	Bra_cxA03g047820.2	B_1_	211	22.71	9.60	43.75	50.00	−0.754	Nucleus
*BcDOF15*	Bra_cxA03g043130.1	C_2.1_	369	40.53	8.25	50.64	50.16	−0.849	Nucleus
*BcDOF16*	Bra_cxA03g027300.1	A	206	22.42	9.25	59.97	38.40	−0.975	Nucleus
*BcDOF17*	Bra_cxA03g022710.1	A	294	31.55	7.65	51.86	44.46	−0.77	Nucleus
*BcDOF18*	Bra_cxA03g021610.1	D_2_	242	25.33	8.45	41.85	54.09	−0.389	Nucleus
*BcDOF19*	Bra_cxA03g020520.1	C_2.2_	238	26.05	5.09	56.69	53.28	−0.578	Nucleus
*BcDOF20*	Bra_cxA03g017030.1	C_3_	148	17.03	9.74	47.59	46.08	−1.092	Nucleus
*BcDOF21*	Bra_cxA03g014790.1	C_2.1_	343	37.27	7.65	49.10	48.92	−0.833	Nucleus
*BcDOF22*	Bra_cxA04g037440.1	C_2.1_	322	35.87	9.04	45.60	41.55	−0.957	Nucleus
*BcDOF23*	Bra_cxA04g021620.1	D_1_	482	52.17	5.95	57.47	53.71	−0.774	Nucleus
*BcDOF24*	Bra_cxA04g012380.1	C_1_	286	31.37	8.44	53.64	53.81	−0.783	Nucleus
*BcDOF25*	Bra_cxA04g012070.1	B_1_	344	36.90	9.25	46.06	55.03	−0.703	Nucleus
*BcDOF26*	Bra_cxA04g008340.1	D_1_	168	18.78	9.48	42.02	67.38	−0.586	Nucleus
*BcDOF27*	Bra_cxA04g006160.1	B_1_	309	33.16	9.24	55.00	48.64	−0.697	Nucleus
*BcDOF28*	Bra_cxA04g000730.1	C_2.1_	376	41.12	8.91	49.07	46.86	−0.851	Nucleus
*BcDOF29*	Bra_cxA05g043670.1	C_2.1_	364	39.91	9.18	53.94	50.82	−0.800	Nucleus
*BcDOF30*	Bra_cxA05g037340.1	B_1_	325	34.74	9.17	53.22	53.48	−0.677	Nucleus
*BcDOF31*	Bra_cxA05g026170.1	A	185	20.85	8.94	68.74	51.14	−1.001	Nucleus
*BcDOF32*	Bra_cxA05g017550.1	A	195	21.23	9.22	57.87	37.54	−1.062	Nucleus
*BcDOF33*	Bra_cxA06g029800.1	C_2.2_	253	28.56	4.73	57.18	52.45	−0.829	Nucleus
*BcDOF34*	Bra_cxA06g027140.1	D_1_	426	46.82	8.68	49.69	49.25	−0.865	Nucleus
*BcDOF35*	Bra_cxA06g019290.1	D_1_	273	31.09	8.59	41.76	57.11	−0.842	Nucleus
*BcDOF36*	Bra_cxA06g018690.1	C_1_	368	39.37	8.03	40.91	56.47	−0.6	Nucleus
*BcDOF37*	Bra_cxA06g016190.1	B_2_	306	34.00	8.69	46.22	51.93	−0.864	Nucleus
*BcDOF38*	Bra_cxA07g032410.1	D_1_	177	19.61	9.53	57.14	63.45	−0.607	Nucleus
*BcDOF39*	Bra_cxA07g031960.1	B_2_	327	35.38	9.59	58.77	57.25	−0.615	Nucleus
*BcDOF40*	Bra_cxA07g028290.1	C_2.2_	262	29.43	4.86	68.35	54.73	−0.71	Nucleus
*BcDOF41*	Bra_cxA07g026600.1	D_2_	220	23.30	9.1	44.34	54.09	−0.424	Nucleus
*BcDOF42*	Bra_cxA07g023660.2	D_1_	413	45.48	7.09	58.74	54.82	−0.759	Nucleus
*BcDOF43*	Bra_cxA07g013090.1	D_1_	386	42.68	9.00	56.75	46.76	−0.868	Nucleus
*BcDOF44*	Bra_cxA07g008880.1	D_1_	368	40.69	8.91	54.12	48.48	−0.833	Nucleus
*BcDOF45*	Bra_cxA08g033840.1	A	200	21.85	9.11	66.52	49.75	−0.829	Nucleus
*BcDOF46*	Bra_cxA08g020800.1	C_3_	254	29.01	7.21	48.56	46.77	−1.033	Nucleus
*BcDOF47*	Bra_cxA08g014180.1	C_2.1_	352	38.32	7.69	45.78	52.93	−0.78	Nucleus
*BcDOF48*	Bra_cxA08g011210.1	D_1_	174	19.32	9.48	45.04	61.15	−0.544	Nucleus
*BcDOF49*	Bra_cxA08g010800.1	B_2_	326	35.35	9.29	58.05	60.09	−0.558	Nucleus
*BcDOF50*	Bra_cxA08g009260.1	D_1_	335	37.90	8.04	62.51	52.90	−0.91	Nucleus
*BcDOF51*	Bra_cxA08g002090.1	B_1_	334	35.88	9.49	46.64	60.48	−0.679	Nucleus
*BcDOF52*	Bra_cxA09g063200.1	A	267	28.65	7.13	50.86	40.60	−0.76	Nucleus
*BcDOF53*	Bra_cxA09g062360.1	C_1_	340	36.67	8.32	53.17	54.82	−0.662	Nucleus
*BcDOF54*	Bra_cxA09g061140.1	B_2_	285	31.90	8.88	49.1	48.21	−0.896	Nucleus
*BcDOF55*	Bra_cxA09g060660.1	D_2_	212	22.59	9.2	46.62	54.81	−0.468	Nucleus
*BcDOF56*	Bra_cxA09g055390.1	C_2.1_	338	36.29	8.45	56.32	52.57	−0.702	Nucleus
*BcDOF57*	Bra_cxA09g032030.1	D_1_	175	19.28	9.35	47.28	56.40	−0.691	Nucleus
*BcDOF58*	Bra_cxA09g031340.1	B_2_	297	32.31	9.15	62.02	54.48	−0.65	Nucleus
*BcDOF59*	Bra_cxA09g029010.1	D_1_	357	40.64	7.61	66.16	56.19	−0.709	Nucleus
*BcDOF60*	Bra_cxA09g024410.1	D_2_	222	23.90	8.80	39.05	53.15	−0.518	Nucleus
*BcDOF61*	Bra_cxA09g022470.1	C_2.2_	242	26.89	6.04	45.69	59.59	−0.656	Nucleus
*BcDOF62*	Bra_cxA09g019640.1	B_1_	343	37.57	9.22	53.97	56.03	−0.741	Nucleus
*BcDOF63*	Bra_cxA09g014630.1	C_2.1_	327	36.32	9.27	44.17	46.82	−0.865	Nucleus
*BcDOF64*	Bra_cxA09g002940.1	B_1_	317	34.61	9.38	44.60	55.08	−0.884	Nucleus
*BcDOF65*	Bra_cxA10g027950.1	D_2_	223	23.34	6.58	53.75	58.25	−0.462	Nucleus
*BcDOF66*	Bra_cxA10g016080.1	C_1_	258	27.91	8.88	55.37	42.79	−0.704	Nucleus
*BcDOF67*	Bra_cxA10g000650.1	B_1_	360	38.45	9.13	59.24	48.28	−0.678	Nucleus

## Data Availability

Data will be made available on request.

## Supplementary Materials

The following supporting information can be downloaded at: https://www.mdpi.com/article/10.3390/biology15211940/s1, Figure S1: Sequence logos of the DOF motifs in *Brassica campestris*. The overall height of each stack reflects sequence conservation measured in bits, whereas the height of individual amino acid letters represents the relative frequency of that residue at the respective position. Figure S2: Identification of different *BcDOF56*-overexpressing lines by PCR and RT-qPCR. (A) Agarose gel electrophoresis of PCR of *Bar* gene in different *Arabidopsis* lines and pCAMBIA3301-BcDOF56 plasmid. WT: wild-type; OE-1, OE-2, OE-3, and OE-4 represent overexpressing *BcDOF56* lines 1, 2, 3, and 4, respectively. (B) RT-qPCR of different *BcDOF56*-overexpressing lines. Bars indicate mean ± SD (*n* = 3), different lowercase above the bars indicate significant differences at *p* < 0.05; Table S1: The primers used in this study; Table S2: Detailed list of orthologous gene clusters between the *DOF* genes in *A*. *thaliana*, *B. campestris*, *B. rapa*, and *B. napus*; Table S3: Detailed list of singleton *DOF* genes in *A*. *thaliana*, *B. campestris*, *B. rapa*, and *B. napus*; Table S4: Detailed summary of gene duplications along with type of duplications in BcDOFs as predicted by MCScanX; Table S5: The Ka/Ks ratios of BcDOF duplication events in *B. campestris*; Table S6: Orthologous gene pairs of *DOF* genes between *B. campestris* and *A. thaliana*, *B. rapa*, and *B. napus*, and the values of Ks; Table S7: Putative *cis*-acting regulatory elements in the promoters of *BcDOFs*.

## Abbreviations

The following abbreviations are used in this manuscript:

ABA	Abscisic acid
CDF	Cycling DOF factor
CDS	Coding sequence
DOF	DNA-binding with One Finger
FPKM	Fragments per kilobase of exon model per million mapped fragments
GAPDH	Glyceraldehyde-3-phosphate dehydrogenase
GFP	Green fluorescent protein
GRAVY	Grand average of hydropathy
HSF	Heat shock factor
HSD	Honestly significant difference
Ka	Nonsynonymous substitution rate
Ks	Synonymous substitution rate
MAPK	Mitogen-activated protein kinase
MDA	Malondialdehyde
MeJA	Methyl jasmonate
ML	Maximum likelihood
MYA	Million years ago
NJ	Neighbor-joining
PEG	Polyethylene glycol
pI	Isoelectric point
RT-qPCR	Reverse transcription quantitative real-time polymerase chain reaction
RNA-seq	RNA sequencing
ROS	Reactive oxygen species
SA	Salicylic acid
SD	Standard deviation
SOD	Superoxide dismutase
TBARS	Thiobarbituric acid reactive substances
UTR	Untranslated region
VDOF	VASCULAR-RELATED DOF
WGT	Whole-genome triplication
WT	Wild-type
XND1	XYLEM NAC DOMAIN 1
Zf-DOF	Zinc-finger DNA-binding with one finger
