# Supplementary material for: Systematic Analysis of the DOF Gene Family in Flowering Chinese Cabbage (Brassica campestris) and the Role of BcDOF56 in Abiotic Stress Adaptation
Source: Biology (Basel). 2026 Oct 25;15(21):1940. doi: 10.3390/biology15211940 (PMC13762410; doi:10.3390/biology15211940)
Supplement: Supplementary file 1 [file biology-15-01940-s001.zip › Figure S1-S2.pdf]

Systematic Analysis of the DOF Gene Family in Flowering Chinese Cabbage (*Brassica campestris*) and the Role of BcDOF56 in Abiotic Stress Adaptation

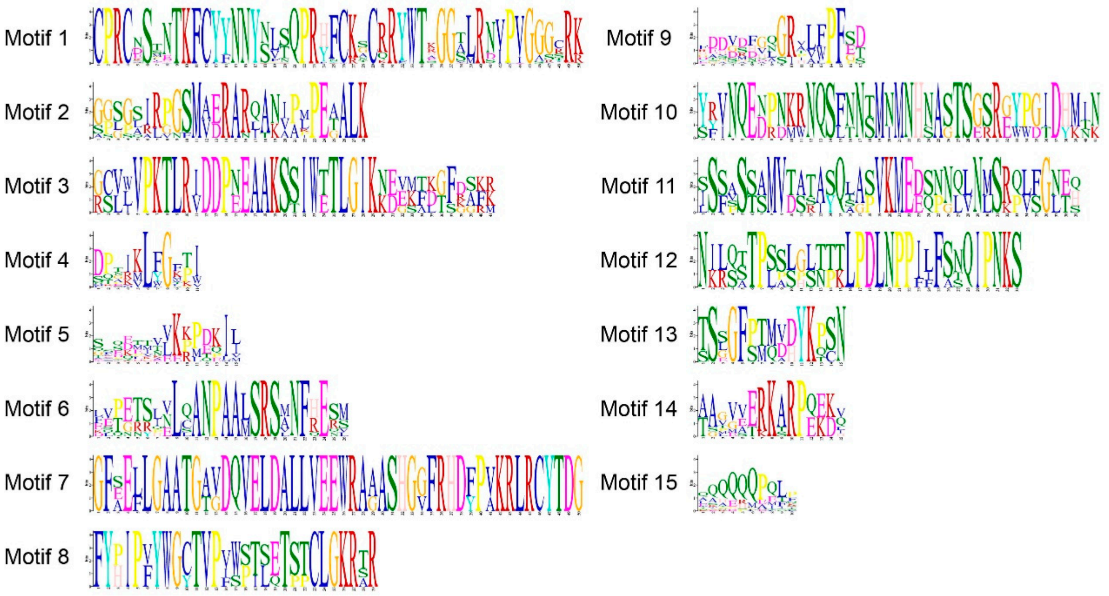

Figure S1: Sequence logos of the DOF motifs in *Brassica campestris.* The overall height of each stack reflects sequence conservation measured in bits, whereas the height of individual amino acid letters represents the relative frequency of that residue at the respective position.

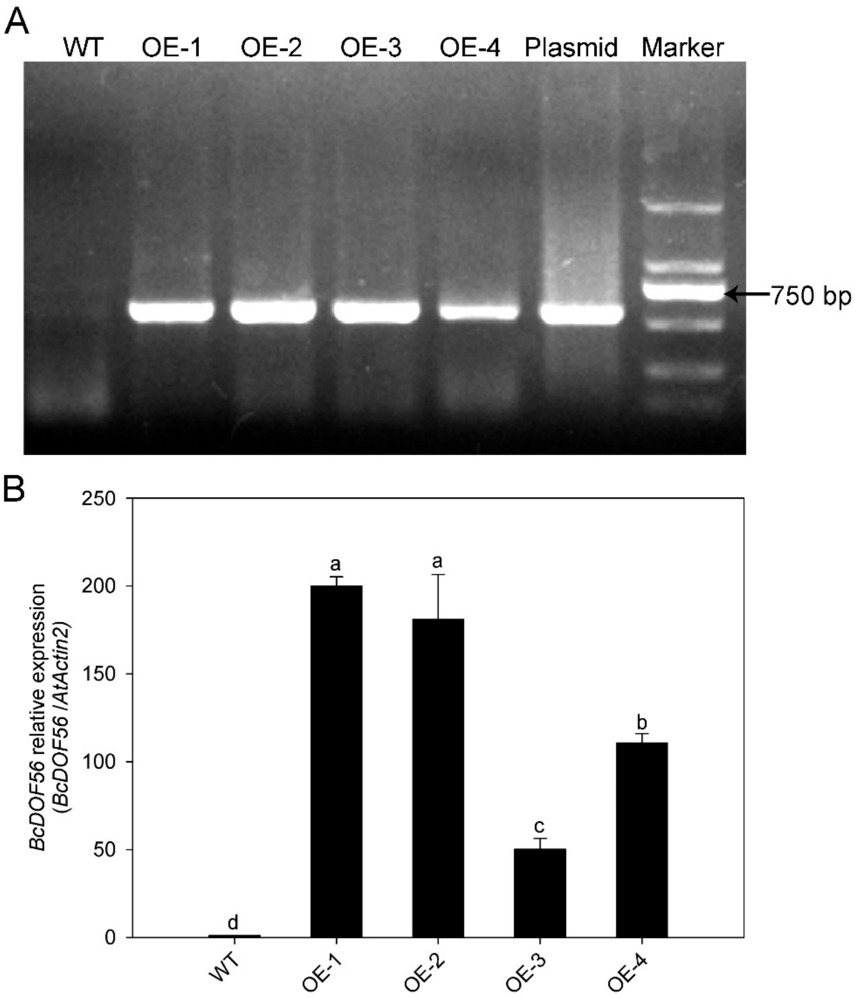

Figure S2. Identification of different *BcDOF56*-overexpressing lines by PCR and RT-qPCR. **(A)** Agarose gel electrophoresis of PCR of *Bar* gene in different *Arabidopsis* lines and pCAMBIA3301-BcDOF56 plasmid. WT: wild-type; OE-1, OE-2, OE-3, and OE-4 represent overexpressing *BcDOF56* lines 1, 2, 3, and 4, respectively. **(B)** RT-qPCR of different *BcDOF56*-overexpressing lines. Bars indicate mean ± SD (n = 3), different lowercase above the bars indicate significant differences at $p < 0.05$.
